# Motivational Disturbances and Effects of L-dopa Administration in Neurofibromatosis-1 Model Mice

**DOI:** 10.1371/journal.pone.0066024

**Published:** 2013-06-10

**Authors:** David F. Wozniak, Kelly A. Diggs-Andrews, Sara Conyers, Carla M. Yuede, Joshua T. Dearborn, Jacquelyn A. Brown, Kazuhiro Tokuda, Yukitoshi Izumi, Charles F. Zorumski, David H. Gutmann

**Affiliations:** 1 Department of Psychiatry, Washington University School of Medicine, St. Louis, Missouri, United States of America; 2 Department Neurology, Washington University School of Medicine, St. Louis, Missouri, United States of America; 3 The Taylor Family Institute for Innovative Psychiatric Research, Washington University School of Medicine, St. Louis, Missouri, United States of America; Max Planck Institute of Psychiatry, Germany

## Abstract

Children with neurofibromatosis type 1 (NF1) frequently have cognitive and behavioral deficits. Some of these deficits have been successfully modeled in *Nf1* genetically-engineered mice that develop optic gliomas (*Nf1* OPG mice). In the current study, we show that abnormal motivational influences affect the behavior of *Nf1* OPG mice, particularly with regard to their response to novel environmental stimuli. For example, *Nf1* OPG mice made fewer spontaneous alternations in a Y-maze and fewer arm entries relative to WT controls. However, analysis of normalized alternation data demonstrated that these differences were not due to a spatial working memory deficit. Other reported behavioral results (e.g., open-field test, below) suggest that differential responses to novelty and/or other motivational influences may be more important determinants of these kinds of behavior than simple differences in locomotor activity/spontaneous movements. Importantly, normal long-term depression was observed in hippocampal slices from *Nf1* OPG mice. Results from elevated plus maze testing showed that differences in exploratory activity between *Nf1* OPG and WT control mice may be dependent on the environmental context (e.g., threatening or non-threatening) under which exploration is being measured. *Nf1* OPG mice also exhibited decreased exploratory hole poking in a novel holeboard and showed abnormal olfactory preferences, although L-dopa (50 mg/kg) administration resolved the abnormal olfactory preference behaviors. *Nf1* OPG mice displayed an attenuated response to a novel open field in terms of decreased ambulatory activity and rearing but only during the first 10 min of the session. Importantly, *Nf1* OPG mice demonstrated investigative rearing deficits with regard to a novel hanging object suspended on one side of the field which were not rescued by L-dopa administration. Collectively, our results provide new data important for evaluating therapeutic treatments aimed at ameliorating NF1-associated cognitive/behavioral deficits.

## Introduction

Neurofibromatosis-1 (NF1) is an autosomal dominant genetic disorder associated with the development of benign and malignant tumors [1]. In addition to tumor predisposition, children with NF1 frequently have learning disabilities, attention deficits and various other cognitive processing disturbances [2], [3], [4]. Understanding the neurological bases of these cognitive and behavioral disturbances in children with NF1 is necessary to develop optimal treatment strategies to ameliorate these problems and enhance the educational achievement and social integration of children with NF1.

In an effort to provide insight into the molecular and cellular mechanisms underlying NF1-associated cognitive and behavioral dysfunctions, we have performed studies involving a strain of *Nf1* genetically-engineered mice that develop optic gliomas (*Nf1* OPG mice). Previously, we demonstrated that *Nf1* OPG mice have mild spatial learning and memory impairments, as well as significant nonselective and selective attention deficits that result from reduced striatal dopamine levels [5]. We have shown that this dopaminergic deficiency in *Nf1* OPG mice is presynaptic in nature and may be quantified by {^11^C}-raclopride positron emission tomography (PET) [6]. We have also demonstrated that a non-selective, exploratory-based attention deficit in *Nf1* OPG mice is corrected by methylphenidate (MPH) and L-Deprenyl, which is associated with the normalization of raclopride binding *in vivo* [6]. Most recently we have used behavioral, electrophysiological and primary culture techniques to demonstrate that reduced dopamine signaling is responsible for some of the defects in neuron function and spatial learning/memory [7]. Results from our studies with *Nf1* OPG mice are consistent with findings from a cohort of children with NF1 which showed that performance on tests of attention and learning were significantly improved following treatment with MPH [8].

In the present study we have extended our functional phenotyping of *Nf1* OPG mice to provide new information on their response to novelty, as well as behavioral deficits related to attention, exploration and olfactory preference. We have also determined whether alterations in hippocampal long-term depression may exist in *Nf1* OPG mice, which might help explain their abnormal behavioral responses to novelty. Lastly, we have assessed whether some of the functional deficits described herein may be ameliorated by administration of L-dopa. Our results suggest that *Nf1* OPG mice exhibit a spectrum of abnormal responses to environmental stimuli which is important for the interpretation of their performance on behavioral tests and for the evaluation of treatments aimed at ameliorating some of the functional disturbances in children with NF1.

## Materials and Methods

### Ethics Statement

All experimental protocols were approved by the Animal Studies Committee of Washington University in St. Louis (protocol nos. 20120110 and 20110111) and are in strict accordance with the NIH Guide for the Care and Use of Laboratory Animals.

### Mice

*Nf1* OPG mice used in the present studies are *Nf1*+/− mice that have a loss of neurofibromin expression in glial (GFAP+) cells (previously referred to as *Nf1*+/−^GFAP^CKO mice). The *Nf1* OPG mutant mice were generated as follows: *Nf1*+/− mice were generated by inserting a pMClneo/poly(A) cassette in the opposite transcriptional orientation into exon 31 of an *Nfl* genomic fragment, providing 7.1 kb of flanking homology at the 5′ end and 1.5 kb of homology at the 3′ end [9] while *Nf1* conditional knockout mice (*Nf1* flox/flox mice) were generated using a similar genomic fragment by inserting LoxP sites flanking exons 31 and 32 [10]. Mice with a neo mouse cassette interrupting the *Nf1* gene (*Nf1*+/− mice) were intercrossed with *Nf1* flox/flox and GFAP-Cre transgenic mice to generate *Nf1* flox/−; GFAP-Cre mice (*Nf1* OPG mice). GFAP-Cre transgenic mice were generated using a 2.2 kb human GFAP promoter to drive expression of a Cre recombinase molecule followed by an internal ribosomal entry site and the beta-galactosidase gene [11]. These mice were maintained on a C57BL/6 background and develop optic gliomas by 3 months of age. Littermate WT controls were used for all experiments. All mice were maintained on *ad libitum* access to food and water and to a 12-h on/12-h off light-dark cycle for all experiments.

### General Experimental Design

**Figure 1:**
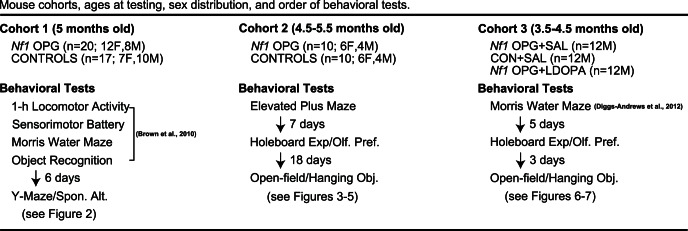
Mouse cohorts used for behavioral tests. Ages of mice at testing, order of behavioral tests and sex distribution for each mouse cohort used in the present study.

The present study was designed to extend and clarify some of the results from our behavioral phenotyping experiments that were conducted in our earlier work with *Nf1* OPG mice [5], which included characterizing the mice as having abnormal exploratory behaviors, mild learning and memory impairments and possible deficits in non-selective and selective attention. Here, we present the results from further testing of a cohort of mice from that study as well as from two other independent cohorts (Figure 1). The cohort from our previously published work (cohort 1) was evaluated on a spontaneous alternation task in a Y-maze at 5 months of age to further investigate possible abnormalities in the response of *Nf1* OPG mice to novelty and/or potential deficits in spatial working memory. This cohort consisted of *Nf1* OPG (n = 20; 12 F, 8 M) and littermate WT control (n = 17; 7 F, 10 M) mice, which had been tested on several behavioral measures as previously reported (Figure 1). Another independent cohort (cohort 2) consisting of *Nf1* OPG (n = 10; 6 F, 4 M) and littermate WT control mice (n = 10; 6 F, 4 M) was assessed on several behavioral tests when they were 4.5–5.5 months of age including: the elevated plus maze to study anxiety-like behaviors and context-dependent effects on ambulatory activity; the holeboard exploration/olfactory preference test to investigate exploratory hole poking in response to novelty and olfactory stimuli; and in an open field to quantify general ambulatory and exploratory behaviors as well as vertical rearing which the mice used to investigate a novel object suspended from one side of the open-field apparatus (Figure 1). Results from a third independent cohort of male mice (3.5–4.5 months old) are also presented here which involved re-assessing the deficits that were observed in the second cohort on the holeboard/olfactory preference and open-field tests, and whether administration of L-dopa to *Nf1* OPG mice was capable of ameliorating these impairments (Figure 1). Thus, the cohort 3 studies involved 3 groups of male littermates: 1) *Nf1* OPG mice treated with saline (*Nf1* OPG+SAL); 2) *Nf1* OPG mice treated with L-dopa (*Nf1* OPG+LDOPA); and 3) WT control mice treated with saline (CON+SAL). This third cohort of mice served as subjects in a portion of our recently published study [7] where they were tested in the Morris water maze following the same injections of L-dopa or normal saline before being evaluated on the behavioral tests described here.

### Behavioral Tests

#### Spontaneous alternation in a Y-maze

Testing was conducted according to our previously published procedures [12]. Briefly, this involved placing a mouse in the center of a Y-maze that contained three arms that were 10.5 cm wide, 40 cm long and 20.5 cm deep where an arm was oriented at 120° with respect to each successive other arm. Mice were allowed to explore the maze for 10 min and entry into an arm was scored only when the hindlimbs had completely entered the arm. An alternation was defined as any three consecutive choices of three different arms without re-exploration of a previously visited arm. Dependent variables included the number of alternations and arm entries along with the percentage of alternations, which was determined by dividing the total number of alternations by the total number of entries minus 2, then multiplying by 100.

#### Elevated plus maze

The elevated plus maze (EPM) testing procedure was similar to our previously described protocol [13]. The apparatus consisted of two opposing open arms (35.0×6.1×0.3 cm) and two opposing enclosed arms (35.0×6.1×15.0 cm) that extended from a central platform (5.5×5.5 cm). The floor and walls of the maze were constructed of black *Plexiglas*. The maze was equipped with photobeam instrumentation (Hamilton-Kinder, LLC, Poway,CA) which allowed for the quantification of time spent, distance traveled, and number of entries made into the open and closed arms and center area. *Nf1* OPG and WT control mice were tested between 8∶00 to 16∶00 hr in a darkened room where the only illumination came from a single 13 W black-light bulb, which simulated “moonlight conditions”. A test began by placing a mouse in an opaque plastic tube and then removing the tube, allowing the mouse to explore the maze. A test session lasted 5 min, and mice were tested over 3 consecutive days.

#### Holeboard exploration/olfactory preference

*Nf1* OPG and WT control mice were evaluated for possible differences in exploratory behaviors using hole poking as a behavioral response, and for olfactory preferences using a protocol similar to our previously published procedures [14], [15], [16]. Our protocol involved the use of a computerized holeboard apparatus (41×41×38.5 cm high), containing 4 corner and 4 side holes, with a side hole being equidistant between the corner holes (Learning Holeboard; MotorMonitor, Kinder Scientific, LLC, Poway, CA). Pairs of photocells were contained within each hole (27 mm in diameter) and were used to quantify the frequency and duration of pokes, whereby a poke that was at least 35 mm in depth was required to be registered as a hole poke. It should be noted that the hole poke response involves a mouse sticking its head into a hole up to and including its’ eyes and is distinguished from more superficial “mini-pokes” which may represent a form of stereotypical behavior [17]. Thus, our term “hole pokes” is distinguished from the term “head dips” which has been used to describe holeboard responses, with the latter term typically referring to a generalized hole poking response, which does not distinguish the depth of the pokes and therefore may include stereotypical behaviors. Odorants were placed at the bottom of diagonally-opposite corner holes although access to the odorants was blocked. A familiar odorant (actual corn cob bedding) and a novel odorant (filter paper impregnated with 2 ml of coconut flavoring; *Durkee*) were used. The other pair of diagonally-opposite holes was empty as were all of the side holes. Holes containing odorants were counterbalanced between and within groups.

#### Open-field activity and response to hanging object

The activity of the *Nf1* OPG and WT control mice was quantified over a 30-min period in an open-field (41×41×38.5 cm high) constructed of Plexiglas and containing computerized photobeam instrumentation (Kinder Scientific, LLC, Poway, CA), whereby the apparatus contained a 16×16 matrix of photocell pairs. The procedure was essentially the same as the one used for our 1-h locomotor activity test [18], [19] except that the test chamber was larger and square rather than rectangular, and activity was analyzed over a 30-min period instead of 60 min to focus on the effects of novelty. Variables related to general activity and exploration (total ambulations, rearing frequency, and rearing time) were analyzed during successive 10-min periods. The next day the mice were placed back into the open field and their rearing in response to investigating a ball (42.7 mm diameter) suspended at the midpoint of a wall of the test chamber so that it was just out of reach of a mouse was quantified over a 10-min period as was rearing in the same area on the opposite side of the field. The wall on which the ball was suspended was counterbalanced within and across groups. Investigation of the ball was quantified using procedures which were similar to those we have used to evaluate object investigation during the object recognition test [5], except that a mouse also reared while investigating the ball. Specifically, an investigative rearing response was scored when mouse reared and directed its head toward the ball with its nose approximately 5 mm or less from the ball. The rearing response was also scored in the same area on the opposite side of the chamber. The total time spent rearing and the number of rears (rearing frequency) were quantified in each area, and the total time spent rearing in all parts of the open field was quantified as well.

### L-dopa Administration

In studies conducted on the third cohort of mice, *Nf1* OPG mice received an intraperitoneal injection of L-DOPA (50 mg/kg; Sigma, St. Louis, MO) dissolved in 2.5 mg/ml ascorbic acid in PBS or a normal (0.9%) saline vehicle, while littermate WT control mice received an injection of normal saline as previously described [7] before being tested 3 hours later on the holeboard exploration/olfactory preference test or on the hanging object measure. Habituation procedures were conducted for both of these tests which did not include drug or saline injections as described above. This dose of L-dopa was used since it rescued the exploratory-based attention system deficits and spatial learning/memory impairments in *Nf1* OPG mice and we wanted to test the efficacy of the same dose to reverse the olfactory preference and investigative rearing deficits in these mice.

### Electrophysiology: Long-term Depression

Long-term depression (LTD) was evaluated using our previously described methods [12]. Briefly, hippocampal slices were prepared from 30-day-old mice, with hippocampi being rapidly dissected, placed in artificial cerebrospinal fluid (ACSF) containing (in mM): 124 NaCl, 5 KCl, 2 MgSO_4_, 2 CaCl_2_, 1.25 NaH_2_PO_4_, 22 NaHCO_3_, 10 glucose, gassed with 95% O_2_–5% CO_2_ at 4–6°C, and sectioned transversely into 400 µm slices. Acutely prepared slices were placed in an incubation chamber containing gassed ACSF for 1 h at 30°C. At the time of study, slices were transferred individually to a submerged recording chamber. Experiments were performed at 30°C with continuous perfusion of ACSF at 2 ml/min. Extracellular recordings were obtained from the CA1 apical dendritic region for analysis of excitatory postsynaptic potentials (EPSPs) using 2 M NaCl glass electrodes with resistances of 5–10 MΩ. Evoked synaptic responses were elicited with 0.2 ms constant current pulses through a bipolar electrode in the Schaffer collateral pathway. Evoked EPSPs were monitored by applying single stimuli every 60 s at an intensity sufficient to elicit 50% maximal EPSPs. After establishing a stable baseline, LTD was induced by applying 1 Hz×900 s low frequency stimulation (LFS) for 15 min. Input-output curves were repeated 20 min and 60 min following 1 Hz stimulation.

### Statistical Analyses

Analysis of variance (ANOVA) models were used to analyze the behavioral data. Repeated measures (rm) ANOVA models containing two between-subjects variables (Genotype and Sex) and one within-subjects (repeated measures) variable (e.g., Time Blocks) were used to analyze most of the behavioral data. The Huynh-Feldt adjustment of alpha levels was utilized for all within-subjects effects containing more than two levels to protect against violations of sphericity/compound symmetry assumptions underlying rmANOVA models. Pairwise comparisons were conducted following appropriate significant over-all effects and were evaluated against Bonferroni correction. One-way ANOVA models and planned comparisons were also used when appropriate.

## Results

### Y-Maze Alternation Performance and Long-term Depression in *Nf1* OPG Mice

**Figure 2:**
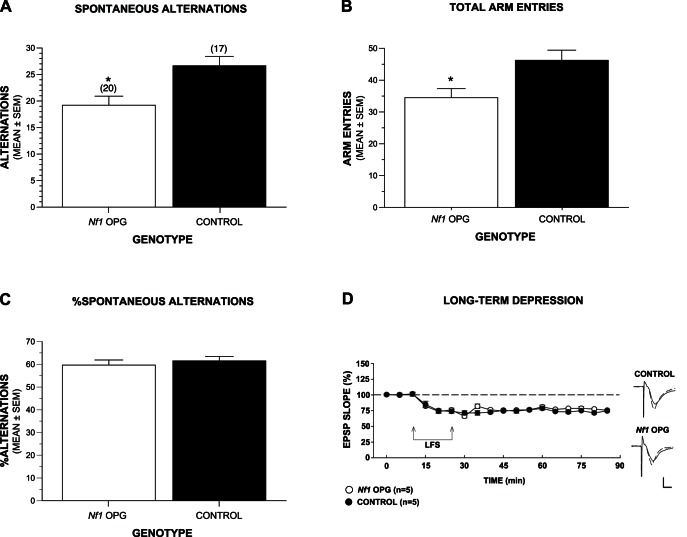
*Nf1* OPG mice show decreased spontaneous alternations in a Y-maze but no deficits in spatial working memory or long-term depression (LTD) in hippocampal slices. (A–B) In cohort 1, *Nf1* OPG mice (n = 20; M = 8; F = 12) made significantly fewer alternations (A; *p = 0.008) and arm entries (B; *p = 0.011) compared to WT control mice (n = 17; M = 10; F = 7) suggesting a diminished response to novelty in the *Nf1* mutant mice. (C) To control for differences in activity, alternation scores were transformed with reference to the number of arm entries in calculating the percentage of spontaneous alternations, and no significant differences in performance were observed between groups suggesting intact spatial working memory in the *Nf1* OPG mice. The mice in cohort 1 were 5 months of age. (D) The graph shows the time course of change in EPSP slope in response to 900 pulse LFS delivered at 1 Hz (connected arrows). LFS produced robust LTD in hippocampal slices from both WT control and *Nf1* OPG mice. Traces to the right of the graph show representative EPSPs from control and *Nf1* OPG slices during baseline (dashed traces) and 60 min following LFS (solid traces). Scale = 1 mv, 5 ms.

We previously reported that *Nf1* OPG mice exhibited mild spatial reference memory deficits in the Morris water maze [5], and we further explored their spatial learning/memory capabilities and response to novelty in the same cohort of mice by evaluating their spontaneous alternation performance. Spontaneous alternation is a measure of exploratory behavior in response to novel environmental stimuli that is dependent on spatial (working) memory capabilities and an optimal level of anxiety [20]. Analysis of the data showed that the *Nf1* OPG mice emitted significantly fewer alternations compared to WT controls (Figure 2A), and they also made significantly fewer arm entries (Figure 2B), thus documenting a diminished exploratory response to novel environmental stimuli in the *Nf1* OPG mice, (Genotype effects: F(1,33) = 8.11, p = 0.008, and F(1,33) = 7.24, p = 0.011, respectively). Since decreased alternations may also reflect spatial working memory impairments, we evaluated this possibility by transforming alternation scores with reference to the number of arm entries made in each mouse to calculate the percentage of spontaneous alternations (Figure 2C). When the levels of exploratory behavior were controlled in this way, no significant differences were observed between groups (Table S1) suggesting that, based on preliminary evidence, *Nf1* OPG mice do not have impaired spatial working memory. No significant effects involving Sex were found for any of the analyses involving the spontaneous alternation data (see Table S1 for all ANOVA effects pertaining to these variables).

Long-term depression (LTD) was evaluated in a separate set of *Nf1* OPG and WT mice since it is a form of hippocampal synaptic plasticity that appears to be important for novelty acquisition [21], [22] and spatial working memory formation, the magnitude of which has been reported to be correlated with spontaneous alternation performance in a Y-maze [23]. In hippocampal slices from both WT control and *Nf1* OPG mice, 1 Hz×900 pulse LFS of the Schaffer collaterals resulted in a persistent depression of EPSPs in the CA1 region for both groups (EPSP change 60 min after LFS = −24.3±1.7%, n = 5, and −23.4±1.1%, n = 5, respectively), thus documenting that the *Nf1* OPG mice did not have deficits in LTD (Figure 2D).

### *Nf1* OPG Mice Show Context-dependent Differences in Activity-related Variables in the Elevated Plus Maze

**Figure 3:**
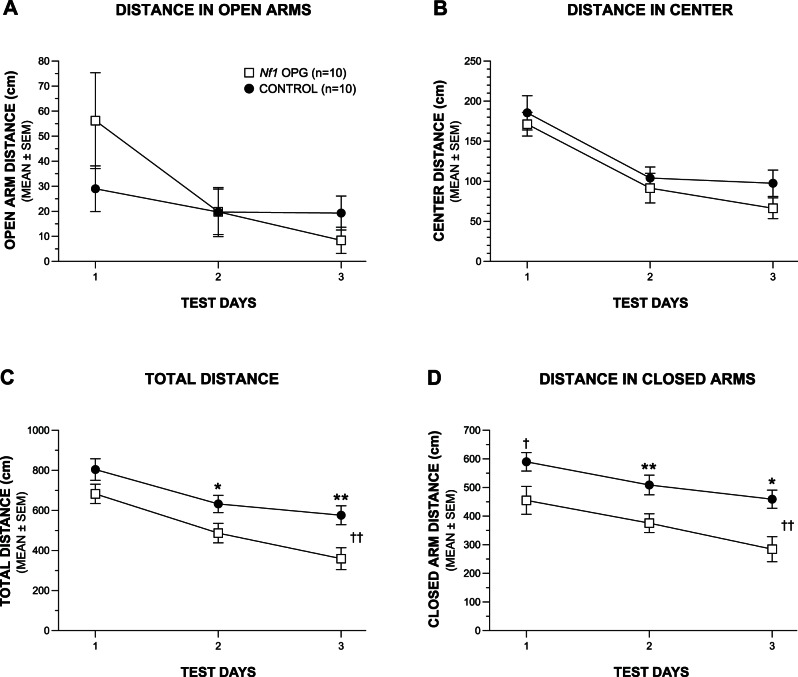
*Nf1* OPG mice display context-dependent alterations in activity in the elevated plus maze (EPM). No differences were observed in the distances traveled by the *Nf1* OPG mice compared to the WT littermate control group from cohort 2 (4.5 months old) in either the open arms (A) or in the center area of the EPM (B). (C) However, the *Nf1* OPG mice traveled a significantly shorter total distance throughout the entire EPM compared to the control group (Genotype effect: ^††^p = 0.009) with significant differences between groups occurring on Test Days 2 (*p = 0.015) and 3 (**p = 0.009). (D) The differences in total distance traveled were found to be mostly due to differences between the two groups in distance traveled in the relatively non-threatening closed arms. Specifically, the *Nf1* OPG mice, on average, traveled a significantly shorter distance in the closed arms compared to the WT controls (Genotype effect: ^††^p = 0.0015), with significant differences being found on Test Days 2 (**p = 0.003) 3 (*p = 0.006), although large differences were also found on Test Day 1 as well (^†^p = 0.024). For both groups in cohort 2 the sample sizes were the same (n = 10), as was the sex distribution (M = 4; F = 6).

Besides having mild spatial learning/memory deficits in the water maze, we previously reported that *Nf1* OPG mice showed evidence of altered emotionality as indexed by their general reluctance to go into the center of the test field during a 1-h locomotor activity test [5]. These observations prompted us to assess anxiety-like behaviors in a second, independent cohort of *Nf1* OPG mice in the elevated plus maze (EPM). Analysis (rmANOVAs) of the classic variables associated with anxiety-like behaviors in the EPM such as distance traveled (Figure 3A), entries made, and time spent in the open arms (Figure S1A–B) did not yield any significant overall effects involving Genotype (see Tables S1–S2 for all EPM ANOVA effects). We also analyzed these three variables after normalizing the values to reflect percentages calculated out of the totals measured in both sets of arms (Figure S1C–E), and did not find any significant overall effects involving Genotype with one exception. The one exception was a significant Genotype by Test Day interaction for the percent of open arm entries made out of the total number of entries for both sets of arms, (F(2,32) = 4.31, p = 0.024). Subsequent pair-wise comparisons showed that this effect was mostly due to differences observed during Test Day 3 (F(1,16) = 6.42, p = 0.022) when the WT control mice made a greater percentage of entries into the open arms out of the total arm entries (Figure S1E), while no differences were observed for Test Days 1 and 2, (F(1,16) = 0.70, p = 0.41 and (F(1,16) = 0.90, p = 0.36, respectively).

Although the EPM data generally did not support the hypothesis that *Nf1* OPG mice exhibited higher levels of anxiety-like behaviors in the maze compared to WT controls, we did find differences in general activity levels that were dependent on the “context” of where the activity was measured in the apparatus. For example, we found that the distance traveled was not different between groups when it was measured in areas of the maze that are traditionally viewed as anxiety-inducing such as the open arms or the central area (Figure 3A–B). However, when the total distance traveled in the EPM was analyzed, robust differences were observed with the *Nf1* OPG mice showing a significantly lower level compared to the WT control group on this variable, (F(1,16) = 8.74, p = 0.009; Figure 3C). This observation is similar to the reduced levels of general ambulatory activity in *Nf1* OPG mice displayed during a 1-h locomotor activity test as previously reported (Brown et al., 2010). Pair-wise comparisons revealed that the total distance traveled by the *Nf1* OPG mice was significantly decreased compared to WT controls for Test Days 2 (p = 0.015) and 3 (p = 0.009). Analyzing the distance traveled in the closed arms showed that the significant differences between groups for the total distance traveled in the EPM were greatly influenced by differences in the distance traversed in the relatively “safe” confines of the closed arms (Figure 3D). Specifically, an rmANOVA of the closed arm distance data yielded a significant main effect of Genotype, (F(1,16) = 14.60, p = 0.0015), and subsequent pair-wise comparisons showed significantly decreased distances on Test Days 2 (p = 0.003) and 3 (p = 0.006) on the part of the *Nf1* OPG mice, with large differences also being observed on Test Day 1 (p = 0.024). No significant overall sex effects were found for any of these EPM analyses (Table S2). Graphs of the data (Figure 3A & D) show that the lack of differences in distance traveled between groups in the open arms of the maze was mostly due to the WT control mice reducing their activity levels to that of the *Nf1* OPG group when compared to the levels observed in the closed arms. These findings suggest that the WT control mice were more sensitive to changes in environmental context with regard to its effects on general ambulatory activity compared to the *Nf1* OPG group. In summary, *Nf1* OPG mice exhibited reduced levels of general ambulatory activity compared to WT controls when measured in contexts which were nonthreatening (closed arms), although these differences disappeared when activity was measured in contexts believed to be anxiety-inducing (open arms and center area).

### *Nf1* OPG Mice Show Abnormal Exploratory Hole Poking Behaviors in Response to Novelty

**Figure 4:**
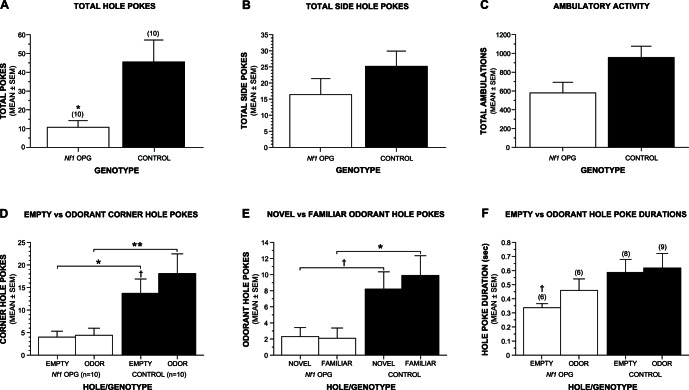
*Nf1* OPG mice exhibit decreased hole poking in response to a novel holeboard apparatus. (A) General exploratory hole poking was significantly attenuated in *Nf1* OPG mice relative to controls in cohort 2 concerning total hole pokes (Genotype effect: *p = 0.023), although differences in total side hole pokes were also large but not significant (B; p = 0.066). (C) *Nf1* OPG mice also showed a trend toward decreased general ambulatory activity although this difference was also not significant (p = 0.053). (D) Analysis of hole pokes made into the corner holes where a familiar (fresh bedding) and a novel (coconut extract) odorant were contained in opposite corner holes while the other two corner holes were empty, revealed the greatest differences between the groups (Genotype effect: p = 0.015). Pair-wise comparisons indicated significantly decreased hole poking by the *Nf1* OPG mice for both the empty (*p = 0.023) and odorant-containing (**p = 0.018) corner holes compared to WT controls. Also, planned comparisons conducted within each group showed that the WT control mice poked less often into the empty versus odorant-containing corner holes (^†^p = 0.046) while the *Nf1* OPG mice did not. (E) In general, the *Nf1* OPG mice poked significantly less often into the odorant-containing holes, (Genotype effect: p = 0.018), where differences were greatest for the hole containing the familiar odorant (*0.024), although large differences were also observed for the novel odorant-containing hole (^†^p = 0.048). Neither group showed a significant preference for either odorant. (F) Planned comparisons conducted within each group indicated that *Nf1* OPG mice had longer poke durations for the odorant-containing holes relative to the empty holes (^†^p = 0.042), while no differences were found in the control mice. During testing, the two groups in cohort 2 were 5.0 months old and had the same sample sizes and sex distribution (n = 10: M = 4; F = 6).

To determine whether *Nf1* OPG mice exhibit abnormalities in other exploratory behaviors that involve a response to novelty (hole poking) that does not rely on the motor systems operative in vertical rearing, the mice were tested on a holeboard exploration/olfactory preference test. The responses of the mice to olfactory stimuli placed in the holeboard were also quantified to determine if *Nf1* OPG mice responded similarly to novel and familiar sensory stimuli. Indices of general exploratory hole poking were found to be significantly decreased in *Nf1* OPG mice (Figure 4A) in terms of total hole pokes (F(1,16) = 6.35, p = 0.023), although differences in total side pokes were large (p = 0.066) but not significant (Figure 3B). General ambulatory activity was also assessed during the task (Figure 3C), and although *Nf1* OPG mice showed a trend toward being less active than WT controls (p = 0.053), the differences were not significant (Table S3 for all ANOVA effects).

An rmANOVA conducted on pokes made into empty and odorant-containing corner holes (Figure 4D) showed that much of the significant Genotype effect with regard to total hole pokes was due to significant differences in the frequency of corner hole pokes, (Genotype effect: F(1,16) = 7.41, p = 0.015). Subsequent pair-wise comparisons showed significantly decreased hole poking by the *Nf1* OPG mice for both the empty (F(1,16) = 6.37, p = 0.023) and the odorant-containing (F(1,16) = 6.89, p = 0.018) corner holes compared to WT controls. In addition, planned comparisons conducted within each group showed that the WT control mice poked less often into the empty versus odorant-containing holes (F(1,16) = 4.69, p = 0.046) but the *Nf1* OPG mice did not show this differential effect (F(1,16) = 0.13, p = 0.72). An rmANOVA was also conducted on the number of pokes made into the novel and familiar odorant-containing holes (Figure 4E). This analysis also yielded a significant effect of Genotype, (F(1,16) = 6.89, p = 0.018), while pair-wise comparisons showed that *Nf1* OPG mice poked less often than WT controls into both the familiar (F(1,16) = 6.23, p = 0.024) and novel (F(1,16) = 4.60, 0.048) odorant-containing holes. However, neither the WT control or *Nf1* OPG groups showed a significant preference for either odorant, (F(1,16) = 0.91, p = 0.35 and F(1,16) = 0.009, p = 0.93, respectively). The duration of hole pokes was also calculated to assess whether *Nf1* OPG mice processed empty and odorant-containing holes differently compared to WT littermate controls. No significant effects involving Genotype were found following an rmANOVA conducted on the poke durations for empty and odorant-containing holes (Figure 4F; Table S3 for ANOVA effects). However, planned comparisons conducted within each group showed that *Nf1* OPG mice had longer poke durations for the odorant-containing holes versus the empty holes (F(1,9) = 5.64, p = 0.042), while smaller differences were observed in the control mice, (F(1,9) = 3.80, p = 0.083). There were no significant overall effects involving Sex for any of the poke frequency or duration variables or for general ambulatory activity (Table S3).

### *Nf1* OPG Mice Exhibit Abnormal Responses to Novelty in an Open Field

**Figure 5:**
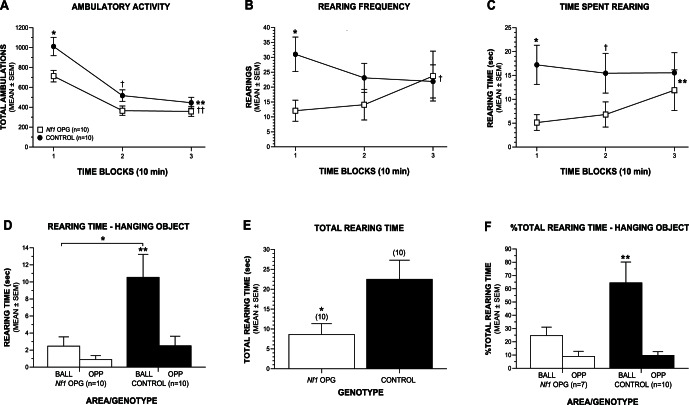
*Nf1* OPG mice exhibit an abnormal response to novel environmental stimuli in an open field. (A) In the cohort 2 mice, locomotor and exploratory activity were quantified over a 30-min period in an open field. An rmANOVA and pair-wise comparisons revealed that *Nf1* OPG mice showed significantly (beyond Bonferroni correction: p<0.017) reduced total ambulations (whole body movements) compared to WT littermate controls but only during the first 10-min block of the open-field test (*p = 0.015), although large differences were also observed during Block 2 (^†^p = 0.038) (Genotype effect: ^††^p = 0.027; Genotype by Time interaction: ^††^p = 0.033). (B) Similarly, *Nf1* OPG mice exhibited significantly decreased numbers of vertical rearings during only the first 10-min time block as well (*p = 0.010). (Genotype by Time interaction: ^†^p = 0.043). (C) The *Nf1* OPG mice also spent significantly less total time rearing in the open field compared to control mice (*p = 0.005) during the first time block with large differences also being observed for the second time block (^†^p = 0.046), (Genotype effect: F(1,16) = 5.00, **p = 0.040). (D) *Nf1* OPG mice displayed significantly reduced rearing to investigate an object (ball) suspended on one side of the open field apparatus relative to control mice (*p = 0.014) although the groups did not differ in the time spent rearing in the same area on the opposite side of the field. In addition, the WT control mice showed significantly increased rearing times to investigate the ball relative to the amount of rearing time displayed on the opposite side of the field (BALL vs OPP; **p = 0.0001), while no significant differences were found in terms of the rearing times between the two areas in *Nf1* OPG mice. (E) *Nf1* OPG mice spent significantly less time rearing in the open field in general (*p = 0.014) compared to the control group. (F) When rearing to investigate the hanging object and rearing displayed in the same area on the opposite side of the field were calculated as percentages of the total rearing time, the WT control mice, but not the *Nf1* OPG mice, showed significant differences in rearing to investigate the ball versus rearing on the opposite side of the field (BALL vs OPP; **p = 0.001). During the open-field testing, the cohort 2 groups were 5.5 months old and consisted of the same sample sizes and sex distribution (n = 10: M = 4; F = 6).

Ambulatory activity and exploratory vertical rearing of the mice were quantified over a 30-min period in an open-field to determine their response to a novel environment. The open-field testing also served to habituate the mice to the apparatus before their levels of investigative rearing were assessed in response to the appearance of a novel object on the following day. An rmANOVA conducted on total ambulations (whole body movements) during the open-field test yielded a significant main effect of Genotype (F(1,16) = 5.93, p = 0.027), and Genotype by Time interaction (F(2,32) = 3.82, p = 0.033), showing that, in general, the *Nf1* OPG mice exhibited significantly reduced ambulatory activity (Figure 5A) compared to WT littermate controls but this differed as a function of time. Subsequent pair-wise comparisons conducted for each 10-min time block showed that the *Nf1* OPG group exhibited significantly decreased activity only during the first 10-min time block, (p = 0.015), relative to control mice although large differences were also observed during the second block, (p = 0.038). A significant Genotype by Time interaction (F(2,32) = 3.47, p = 0.043) was also found with regard to vertical rearing frequency, a variable considered to be a measure of nonselective attention (Figure 5B). Pair-wise comparisons showed that the *Nf1* OPG mice had significantly reduced rearing relative to controls only during the first 10-min time block (p = 0.010). The *Nf1* OPG mice also spent significantly less time rearing compared to the WT controls, (F(1,16) = 5.00, p = 0.040), with subsequent comparisons showing significant differences during the first 10-min block (p = 0.005), while large differences were also observed during the second 10-min block (p = 0.046; Figure 5C). No significant effects involving Sex were found for any of the above variables (Table S4).

The differences in rearing behaviors indicate that *Nf1* OPG mice may not respond normally to the general features of a novel environment suggesting nonselective attention deficits on the part of the *Nf1* OPG mice. To determine whether *Nf1* OPG mice also exhibit more selective attention disturbances to specific novel stimuli, they and WT control mice were placed back into the open field 24-h later and their rearing to investigate an object (a small ball) suspended from one side of the apparatus, which was placed just out of reach, was quantified over a 10-min period. An rmANOVA conducted on the amount of time the mice engaged in rearing to investigate the hanging ball versus the rearing time exhibited in the same area on the opposite side of the test chamber (without a ball), revealed a significant main effect of Genotype, (F(1,16) = 6.34, p = 0.023; Figure 5D), and Genotype by Area interaction, (F(1,16) = 8.59, p = 0.010). Pair-wise comparisons showed that this effect was due to *Nf1* OPG mice spending significantly less time rearing to investigate the hanging ball (p = 0.014) relative to WT controls, although no differences between groups were observed for rearing on the opposite side of the chamber (Table S5). In addition, the control mice spent significantly more time rearing in response to the ball versus the same area on the opposite side of the field, (F(1,16) = 25.23, p = 0.0001), but the *Nf1* OPG mice did not. We also analyzed rearing frequency as an index of investigative behavior. Although analysis of this variable did not yield a significant effect involving Genotype (Table S5), we conducted planned comparisons within each group based on the rearing duration findings. The results from this anlaysis showed that the WT control mice reared significantly more often to investigate the hanging object compared to rearing on the opposite side of the field (F(1,16) = 15.10, p = 0.001), while the *Nf1* OPG group did not display differences in rearing frequency between the two areas (Figure S1F). Similar to the results from the other tests, no significant overall effects involving Sex were found regarding the variables from the hanging object task (see Table S5 for all ANOVA effects for hanging object variables).

Although the within-subjects (repeated measures) comparisons conducted in each group (described above) utilized only the rearing levels exhibited in each group as a reference point for the analyses, we were still concerned that the low level of rearing in the *Nf1* OPG group may have biased the data. As a result we conducted additional analyses to further evaluate this possibility. As suspected, the total rearing time (Figure 5E) exhibited by the *Nf1* OPG mice throughout the entire test field was significantly reduced compared to the littermate WT control group (F(1,16) = 7.70, p = 0.014). Considering this finding, we further analyzed the investigative rearing data by expressing the time spent rearing to the ball and opposite area as percentages of the total time spent rearing in the test field (Figure 5F). Even with this “normalization” of the data, the control mice still showed over 2.5 times greater percentages in rearing to investigate the ball compared to the *Nf1* OPG mice, although an rmANOVA of the data did not yield any significant overall effects involving Genotype. However, planned comparisons conducted within each group showed that the WT control mice had significantly greater percentages of rearing to the ball versus the percentages shown in the opposite area of the field (F(1,13) = 16.33, p = 0.001), while the *Nf1* OPG showed no differences in rearing percentages across the two areas. It should be noted that the power of these latter analyses was limited compared to the “non-normalized” analyses because three of the *Nf1* OPG mice did not rear at all during the test and the values of their normalized rearing percentages became mathematically undefined. Thus, these three mice were deleted from the overall rmANOVA, and planned comparisons were conducted within each group. Nevertheless, the WT control mice showed robust differences in rearing to the ball versus the levels exhibited on the other side of the field while the *Nf1* OPG group did not. In summary, data from the hanging object test suggest that *Nf1* OPG mice show greatly reduced levels of rearing to investigate a novel object compared to WT controls.

### Effects of Habituation and L-dopa Administration on Exploratory Hole Poking and Olfactory Preference Behaviors in *Nf1* OPG Mice

**Figure 6:**
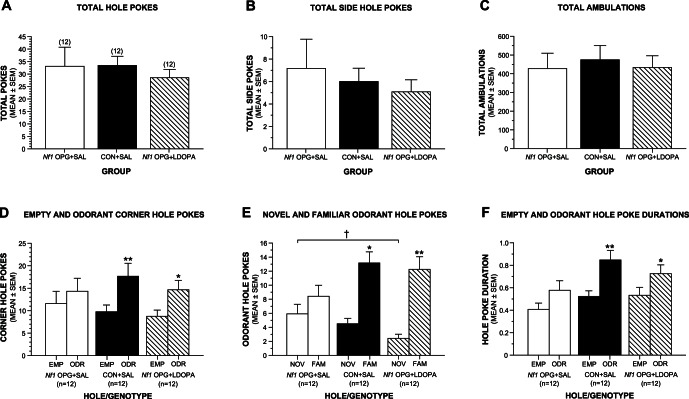
Habituation increases exploratory hole poking in male *Nf1* OPG mice and L-dopa administration rescues normal olfactory preference behaviors. (A–C) In cohort 3, no differences were observed between saline-treated WT control mice (CON+SAL), saline-treated *Nf1* OPG mice (*Nf1* OPG+SAL) or *Nf1* OPG mice treated with L-dopa (*Nf1* OPG+LDOPA) in terms of total hole pokes (A), total side pokes (B), or general ambulatory activity (C). (D) However, planned comparisons showed that the CON+SAL and *Nf1* OPG+LDOPA groups displayed a significant preference (increased poke frequency) for the odorant-containing versus the empty corner holes (EMP vs ODR; **p = 0.003 and *p = 0.020, respectively), while the *Nf1* OPG+SAL mice did not show a significant preference. (E) Similarly, the CON+SAL and *Nf1* OPG+LDOPA mice exhibited a significant preference for the familiar (fresh homecage bedding) versus the novel (coconut) odorant (NOV vs FAM; *p = 0.0001 and **p<0.00005, respectively), in contrast to the *Nf1* OPG+SAL group which again did not show a significant preference. In addition, the *Nf1* OPG+SAL mice poked significantly more often into the hole containing the novel odorant compared to the levels observed in the *Nf1* OPG+LDOPA group (^†^p = 0.016). (F) The average hole poke durations were significantly greater for the odorant-containing versus the empty holes in both the CON+SAL and *Nf1* OPG+LDOPA groups (EMP vs ODR; **p = 0.001 and *p = 0.041, respectively), while the *Nf1* OPG+SAL mice did not show significant differences in poke durations for the different types of holes. The mice in cohort 3 were all males that were 3.5–4.5 months of age and each of the three groups had the same sample size (n = 12).

Behavioral testing was conducted on a third cohort of mice (all males) to assess the effects of L-dopa administration (50 mg/kg) on performance in the holeboard and open-field. In the study described above involving the second cohort, the mice were not habituated to the holeboard apparatus before being tested in order to evaluate their hole-poking behaviors in response to a novel environment. The hole poking of the *Nf1* OPG mice was so low under these conditions that it may not have been possible to provide an adequate assessment of olfactory preference behaviors in these mice. For example, only half of the *Nf1* OPG mice poked into holes containing a familiar or novel odorant while 9/10 WT control mice poked into these holes. To provide a more valid test of olfactory preferences when we assessed the effects of L-dopa on holeboard performance, we habituated the mice to the apparatus before testing them on the following day. Under these conditions, *Nf1* OPG mice treated with saline (*Nf1* OPG+SAL) exhibited similar levels of general hole poking and ambulatory activity during the test trial compared to saline-treated WT controls (CON+SAL) and *Nf1* OPG mice treated with L-dopa (*Nf1* OPG+LDOPA). Specifically, no significant effects involving Group were found following ANOVAs conducted on total hole pokes (Figure 6A), total side pokes (Figure 6B), or total ambulations (Figure 6C) (see Table S6 for all ANOVA effects).

Although groups performed similarly in terms of exploratory hole poking and general ambulatory activity, they displayed differences in olfactory preference behaviors. Moreover, an rmANOVA conducted on the poke frequency data pertaining to the empty versus odorant-containing corner holes (Figure 6D) also revealed no effects involving Group (Table S6). Importantly however, planned comparisons conducted within each group showed that the CON+SAL and *Nf1* OPG+LDOPA mice poked significantly more often into the odorant-containing versus the empty corner holes, (F(1,33) = 10.72, p = 0.003 and F(1,33) = 5.99, p = 0.020, respectively), whereas the *Nf1* OPG+SAL mice did not show a significant preference (Table S6). Differences in olfactory preferences were also found following analysis of the data pertaining to the novel (coconut) versus familiar (fresh homecage bedding) odorants (Figure 6E). Specifically, an rmANOVA yielded a nonsignficant Group effect (Table S6) but a significant Group by Hole (odorant) interaction, (F(2,33) = 4.45, p = 0.019). At least part of this effect was due to *Nf1* OPG+SAL mice poking significantly more often into the novel odorant-containing hole than the *Nf1* OPG+LDOPA group, (F(1,33) = 6.51, p = 0.016). More importantly, planned comparisons indicated that the CON+SAL and *Nf1* OPG+LDOPA groups each showed a robust and significant preference for the familiar versus the novel odorant, (F(1,33) = 21.53, p = 0.0001 and F(1,33) = 27.72, p<0.00005, respectively), but the *Nf1* OPG+SAL mice did not display a significant preference (Table S6). Analysis of the average poke duration data for the empty and odorant-containing holes (Figure 6F) yielded no significant overall effects involving Group (Table S6), although planned comparisons showed that the CON+SAL and *Nf1* OPG+LDOPA mice each exhibited significantly longer poke durations for the odorant-containing versus empty corner holes, (F(1,33) = 12.84, p = 0.001 and F(1,33) = 4.51, p = 0.041, respectively). In contrast, the *Nf1* OPG+SAL mice exhibited a similar trend in preference, but their poke durations for the empty versus odorant-containing corner holes were not significantly different (Table S6).

### Investigative Rearing Deficit in *Nf1* OPG Mice is not Restored by L-dopa

**Figure 7:**
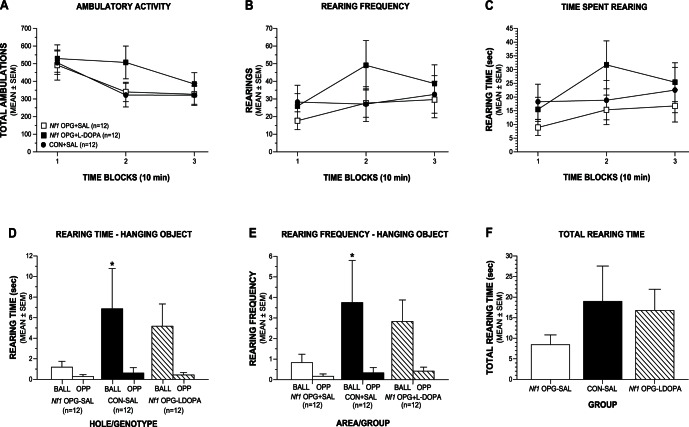
The investigative rearing deficit in male *Nf1* OPG mice is not rescued by L-dopa administration. (A–C) No significant overall effects of Group were found in the third cohort of mice for ambulatory activity (A), rearing frequency (B), or time spent rearing (C) during a 30-min habituation trial in the open-field apparatus, which was conducted the day before the test session and did not include any drug/vehicle injections. (D) In contrast, planned comparisons showed that the CON+SAL mice spent significantly more time rearing to investigate the hanging object (ball) compared to the time spent rearing in the same area at the opposite end of the field (BALL vs OPP; *p = 0.017), while the *Nf1* OPG+SAL group did not show significantly different rearing times with regard to the ball versus the opposite area. The *Nf1* OPG+LDOPA mice reared for substantially longer times in investigating the ball versus the opposite area but these differences were not statistically significant (p = 0.064). (E) Similar results were found for the rearing frequency data where planned comparisons revealed that the CON+SAL mice spent significantly more time rearing to investigate the hanging ball versus the time spent rearing in the opposite area of the field (BALL vs OPP; *p = 0.010), while the *Nf1* OPG+SAL group did not. Again, the *Nf1* OPG+LDOPA mice showed a trend toward greater investigative rearing toward the ball versus the opposite area but these differences were not statistically significant (p = 0.063). (F) Although the *Nf1* OPG+SAL mice tended to spend less time rearing in general throughout the field compared to the CON+SAL and *Nf1* OPG+LDOPA groups, no statistically significant effects were observed for this variable. The male mice in cohort 3 were 3.5–4.5 months old and the sample size for each of the three groups was the same (n = 12).

In an effort to replicate our finding of decreased investigative rearing in *Nf1* OPG mice in the second cohort as well as evaluate the effects of L-dopa on this behavioral deficit, we conducted the same test in the third cohort. For this third cohort, no injections were given before conducting a habituation trial in the open field on day 1, followed by a test trial on day 2 in the presence of the novel hanging object (ball), which occurred 3 h after L-dopa or saline injections. During the habituation trial, the groups performed similarly in terms of ambulatory activity, rearing frequency, and time spent rearing (Table S7), although the *Nf1* OPG+LDOPA mice tended to exhibit increased levels of these variables during the second time block (Figure. 7A–C). However, rmANOVAs yielded no significant main or interaction effects involving Group for any of these variables thus confirming the lack of differences among the groups during the open-field habituation (Table S7). In contrast to these results, there was evidence of different degrees of investigative rearing selectivity within each group during the hanging object test (Figure 7D). For example, although no significant effects involving Group were found, planned comparisons showed that the CON+SAL mice reared for a significantly greater period of time to investigate the ball compared to rearing in the same area in the opposite end of the field, (F(1,33) = 6.36, p = 0.017), while the *Nf1* OPG+SAL mice did not show significantly different rearing times (Table S7). The *Nf1* OPG+LDOPA mice showed a strong trend toward increased rearing to investigate the ball versus the empty area, although this comparison failed to achieve statistical significance (p = 0.064). Analysis of the rearing frequency data revealed the same results (Figure 7E). Specifically, there were no significant effects involving Group (Table S7), although planned comparisons showed that the CON+SAL mice reared significantly more often to investigate the ball compared to rearing in the same area at the opposite end of the field, (F(1,33) = 7.43, p = 0.010), while the *Nf1* OPG+SAL mice did not show significant differences in rearing frequencies (Table S7). Again, the *Nf1* OPG+LDOPA mice showed a strong nonsignficant trend toward increased rearing frequency to investigate the ball versus the empty area (p = 0.063). Lastly, we analyzed the total rearing time exhibited throughout the maze (Figure 6F) and total ambulations (Figure S1G) during the test but found no significant differences among the groups (Table S7). In summary, the CON+SAL mice exhibited significantly more rearing to investigate the hanging object compared to the opposite area in the field, while the *Nf1* OPG+SAL group did not, which is consistent with our previous findings reported above. In addition, although the *Nf1* OPG+LDOPA mice showed more rearing to investigate the hanging object compared to the opposite area, these differences were not statistically significant.

## Discussion

We performed studies with *Nf1* OPG mice to gain a more detailed understanding of the molecular and neurochemical mechanisms underlying the learning disabilities, impaired attention, and other cognitive processing deficits observed in children with NF1 [3], [8]. Our initial characterization of *Nf1* OPG mice included several behavioral anomalies such as mild spatial learning/memory deficits, abnormal exploratory behaviors suggesting impairments in nonselective and selective attention, as well as alterations in emotionality that might impact exploratory-related behaviors [5]. In the present work we have extended the characterization of the behavioral phenotype of *Nf1* OPG mice and thus provide important details for interpreting various behavioral disturbances in these mutant mice, and for developing possible treatment strategies as well.

In previous work, we showed that *Nf1* OPG mice have mild probe trial retention deficits in the water maze and altered performance during the object recognition test [5]. In an extension of that study, we further evaluated those same mice (cohort 1) on the spontaneous alternation task in a Y-maze (SAY) to provide another measure of exploratory behavior in response to a novel environment and to generate preliminary data about whether *Nf1* OPG mice might have spatial working memory deficits. As would be predicted from our previous 1-h locomotor activity results [5], the *Nf1* OPG mice showed significantly reduced numbers of alternations and arm entries, indicating abnormal exploratory behavior in response to a novel environment. However, when these differences were normalized by computing percent alternations, the groups exhibited similar performance levels thus suggesting that spatial working memory may be intact in *Nf1* OPG mice, although additional studies are required to confirm this. The SAY results underscore important caveats when interpreting data from tasks like the object recognition test which are based on the assumption that novel environmental stimuli are equally reinforcing for motivating investigative behaviors across test groups. This is likely not to be the case with *Nf1* OPG and WT control mice which makes it difficult to interpret differences between these two groups with regard to investigation of familiar versus novel objects as well as objects that are in familiar versus novel locations.

Another finding from our earlier work on assessing behavior during a 1-h locomotor activity test was that *Nf1* OPG mice showed signs of altered emotionality based on their reluctance to enter the center of the test field [5]. Our EPM results suggested that *Nf1* OPG mice do not show increased levels of anxiety-like behaviors according to the classic indicators involving behaviors emitted in the open arms. However, the *Nf1* OPG mice displayed reduced levels of ambulatory activity compared to control mice in areas of the maze that were non-threatening (i.e., the closed arms) but exhibited similar activity levels in “less secure” areas of the maze like the open arms and the central area of the maze. Collectively, these results suggest that the environmental context within which activity-related behaviors are measured has a significant impact on the differences observed between *Nf1* OPG and control mice. Preliminary evidence suggests that certain threatening environmental contexts may reduce exploratory behavior in WT control mice to levels seen in the *Nf1* OPG group, such that differences in ambulatory activity (distance traveled) are no longer observed.

In our original work we firmly established that *Nf1* OPG mice exhibit reliable reductions in rearing when responding to novel environments and stimuli compared to control mice [5]. However, more recently we considered the possibility that there may be something idiosyncratic about this specific abnormal exploratory response in these mutant mice, and that other measures of behavioral exploration may be intact. Our finding that *Nf1* OPG mice also showed very robust deficits in hole poking when placed in the novel holeboard environment is consistent with our previous results in that hole poking is also considered a classic exploratory response in rodents [24], although it invokes a very different motor response compared to that involved in vertical rearing. Thus, *Nf1* OPG mice also exhibited an attenuated response to a novel environment using a completely different behavioral response to assess exploratory behavior.

The results from testing cohort 2 in the EPM and holeboard suggested to us that we should also assess exploratory behaviors in an open-field since the context of the apparatus may result in somewhat different findings from those of the original 1-h activity test [5]. Analyses of the data from the open-field test showed that *Nf1* OPG mice exhibited abnormal exploratory responses including decreased ambulatory activity and decreased vertical rearing frequency relative to WT littermate controls but only when the mice were first exposed to a novel open field (first 10 min), but not thereafter. The brief period of time when significant differences were found between the *Nf1* OPG and WT control mice in the open-field were surprising considering the degree of differences in general ambulatory activity and rearing that were observed previously during a 1-h locomotor activity test [5], where significant differences were observed between groups during almost the entire 60-min test. These varying results may be due to the test chamber used in the 1-h locomotor activity test being more similar to the residential home cages of the mice and thus the novel aspects of the environment are likely not as salient as those in the open field, making the latter a better test procedure for evaluating behavioral responses to novelty. Moreover, the lack of similarity of the open field to the home-cage environment of the mice seems to have produced a general dampening of exploratory behaviors compared to levels observed in the 1-h locomotor activity test [5].

To follow up on our holeboard and open-field-related results from testing cohort 2, we evaluated another cohort of male mice and included an *Nf1* OPG group that was treated with L-dopa (50 mg/kg). The holeboard and open-field tests were conducted after these mice had served as subjects in a Morris water maze experiment, where this same dose of L-dopa was found to rescue the retention deficit exhibited by *Nf1* OPG mice during a probe trial. For the holeboard test we included a 30-min habituation trial without any odorants being present or drug/saline injections being given in an effort to increase general hole poking levels to potentially provide more valid assessments of olfactory preference made on the following test day when drug/saline injections were administered. This protocol change resulted in no differences being observed in general hole poking indices or ambulatory activity during the test session. However, the CON+SAL and *Nf1* OPG+LDOPA groups each showed a significant preference for poking into the corner holes containing the odorants versus the empty corner holes while the *Nf1* OPG+SAL mice showed no such preference. Differences were more striking with regard to poking into the hole containing a familiar versus a novel odorant where the CON+SAL and *Nf1* OPG+LDOPA mice displayed a highly significant preference for the familiar odorant-containing hole while the *Nf1* OPG+SAL mice again showed no significant preference. In addition, both the CON+SAL and *Nf1* OPG+LDOPA mice each showed significantly increased average hole poke durations for the odorant-containing versus the empty holes, while the *Nf1* OPG+SAL group did not show an increased hole poke duration for the odorant-containing holes.

Collectively, our holeboard results suggest that *Nf1* OPG mice show reduced exploratory hole poking in response to a novel environment. However, when the experimental protocol was altered by including a habituation trial that preceded the test session, exploratory hole poking in *Nf1* OPG mice increased to levels that were equivalent to those observed in WT control mice, thus allowing olfactory preference behaviors to be studied in a valid manner. Under these conditions, *Nf1* OPG mice exhibited an abnormal olfactory preference compared to control mice, which was rescued by L-dopa administration. Currently, it is not clear how dopamine is related to the abnormal olfactory preference abnormalities in *Nf1* OPG mice. However, it is worth noting that in vertebrates, the olfactory bulb (OB) contains the major dopamine system of the forebrain [25]. Dopaminergic cells in the OB function as interneurons receiving innervation from the primary output neurons of the olfactory bulb (receptor neurons, and mitral and tufted cells), and serve important functions in terms of mediating olfactory discrimination and establishing dynamic ranges of odorant sensory information important for detection [25]. Moreover, olfactory dysfunction occurs as an early “pre-clinical” sign of Parkinson’s disease [26], and evidence is accumulating suggesting that impaired olfaction may serve as a cognitive marker for neuropsychiatric disorders that involve disturbed dopaminergic neurotransmission such as schizophrenia and childhood onset disorders like ADHD [27]. Thus, it is reasonable to consider that the abnormal olfactory preference behaviors in *Nf1* OPG mice may reflect olfactory sensory deficits possibly resulting from dopamine deficiency. Data from additional experiments may help resolve this issue although it will be difficult to do so since *Nf1* OPG mice have documented attention system dysfunction, learning/memory impairments and possible motivational disturbances which might confound the results from sensory-based experiments that rely on these functions to be intact.

It is also important to note that the familiar versus novel dimension of the odorants used in the present experiments is probably less important for determining olfactory preference than other aspects of these stimuli. Specifically, a preference for the odor of familiar bedding has been reported previously by other investigators [28], and we have observed this same preference in several of our own studies involving WT controls for other mutant mouse strains [14], [15], [16]. Based on these findings, we chose the odor of familiar bedding for evaluating olfactory preferences in the present study since it appears to be a reliably-preferred odorant in laboratory mice.

Rearing induced by environmental change has been characterized as an index of non-selective attention in rodents [29], [30]. Within this model, rearing frequency is thought to reflect the degree of orientation to environmental stimuli, while rearing duration represents the time spent scanning the environment and processing information. In the present study we have interpreted the decreased rearing in *Nf1* OPG mice to represent deficits in nonselective attention that occur during early exploration of a novel open field. This interpretation is similar to the conclusions we reached concerning the reduced rearing of *Nf1* OPG mice during a 1-h locomotor activity test [5], although the diminished rearing in the open field observed in the present study seems to be more explicitly related to an attenuated response to novelty. In our previous study [5], we presented evidence from monitoring rearing during an object recognition test which suggested that *Nf1* OPG mice also have selective attention deficits. With that test it was not possible to separate investigative rearing from other behavioral responses during object investigation when mice reared in the vicinity of an object. In the present study, having objects suspended above the floor and just out of reach makes rearing the only possible response for object investigation. Using this procedure, *Nf1* OPG mice showed decreased investigative rearing. Not only did they exhibit reduced rearing to investigate the suspended ball relative to WT controls, but they also showed no differences in rearing to investigate the ball versus the amount of rearing emitted in the same area on the opposite side of the test chamber. In contrast, the control mice showed robust differences with regard to the investigative rearing directed at the ball versus rearing on the opposite side of the chamber.

In an effort to replicate and expand upon the above findings, we conducted an additional experiment in the third cohort of mice that was designed to determine if the investigative rearing deficit in *Nf1* OPG mice could be ameliorated by administering the same dose of L-dopa that was used in our previous 1-h activity, Morris water maze, and holeboard experiments. A 30-min habituation trial in the absence of any drug/saline injections being given was also conducted for this measure, which was followed by the 10-min hanging object test on the following day when L-dopa/saline injections were administered. During habituation, no differences were observed between the groups with regard to general ambulatory activity, vertical rearing frequency or time spent rearing although there was a trend for the *Nf1* OPG+LDOPA mice to exhibit higher levels of all three variables during the second time block. These results are somewhat different from the open-field results in the second cohort where the *Nf1* OPG mice showed decreased activity and rearing during only the first 10-min block. Exposure to several sessions of injections may have dampened the response of the CON+SAL group to the novelty of the open field and thus eliminated differences relative to the *Nf1* OPG+SAL group early on in the test session. Nevertheless, the previously observed investigative rearing deficits in the *Nf1* OPG mice were replicated in the third cohort. Specifically, the CON+SAL mice exhibited significantly greater levels of investigative rearing (time and frequency) to the hanging object compared to the rearing they exhibited at the opposite end of the field, in contrast to the *Nf1* OPG+SAL mice which showed no such differences. The *Nf1* OPG+LDOPA mice also showed large differences in rearing between the hanging object and empty opposite area although these differences were not statistically significant (p<0.065 for both time and frequency).

The lowered levels of investigative rearing in *Nf1* OPG mice may have been influenced by several factors. For example, it is possible that the investigative rearing deficits in *Nf1* OPG mice may have been due to compromised visual function since they were tested after optic gliomas were likely to be present, and their inability to clearly visualize the hanging object may have decreased rearing. This seems unlikely since we have demonstrated that *Nf1* OPG mice are not impaired at this age during the cued condition in the water maze nor do they perform differently from WT controls in terms of their visual acuity as measured by the virtual optomotry technique [5]. If *Nf1* OPG mice have subtle visual deficits at this age, they are not great enough to disrupt important visually-guided behaviors. In the same study we showed that *Nf1* OPG mice did not exhibit any performance impairments at this age on a battery of sensorimotor measures or on the constant speed or accelerating rotarod tests, so it is also unlikely that compromised motor/sensorimotor capabilities were responsible for the reduced rearing. After considering these issues, a more reasonable interpretation of the investigative rearing data is that *Nf1* OPG mice exhibit a form of selective inattention to novel objects placed in their environment.

It is reasonable to question whether differences in the ages of the three cohorts used in the present study as well as differences in test sequences may have affected the behavioral results. The three cohorts were composed of young adult mice that were 3.5 to 5.5 months old, a range which does not represent a large disparity in age. In addition, different test sequences were used in cohorts 2 and 3 concerning the holeboard exploration/olfactory preference and open-field/hanging object tests. Specifically, in cohort 2 both tests followed the EPM, while in cohort 3, both tasks followed water maze testing. Despite the differences in age and test sequences, deficits in olfactory preference and investigative rearing in *Nf1* OPG mice were replicated across the two cohorts thus documenting the reliability of these disturbances and the lack of confounding influences of age and test sequence.

One might also question whether the behavioral disturbances in *Nf1* OPG mice reported here could be parsimoniously explained by the differences in locomotor activity and/or spontaneous behaviors between the *Nf1* OPG and WT control groups. The results in the present study suggest that it is an oversimplification to posit these differences as explanations for the variety of behavioral disturbances observed in these mutant mice. First, our EPM results demonstrate that there are environments in which *Nf1* OPG mice do not show reduced levels of activity and/or exploratory behaviors relative to WT controls. Our EPM data suggest that the environmental context (threatening vs. non-threatening) may be important for determining whether differences are observed between *Nf1* OPG and WT control mice in ambulation and exploratory behaviors, thus suggesting altered emotionality on the part of the *Nf1* OPG mice. Secondly, *Nf1* OPG mice exhibit reductions in other exploratory behaviors such as hole poking when there are no differences in ambulatory activity. Our data also show that familiarizing the mice with the holeboard apparatus by conducting a habituation trial before testing, results in equalizing the levels of general exploratory hole poking between *Nf1* OPG and WT control mice but also produces differences in hole poking related to olfactory preference. It is difficult to explain these results by only referring to differences in general activity and/or spontaneous movements between the groups. In addition, *Nf1* OPG mice show investigative rearing deficits during the hanging object test even when the data are normalized to controls for differences in general rearing levels within the test field. In summary, our results suggest that *Nf1* OPG mice have a variety of motivational disturbances that have a significant impact on several of their behaviors, some of which may be rescued by L-dopa administration.

In the current report, we have presented evidence demonstrating that *Nf1* OPG mice exhibit an abnormal response to novelty, particularly as it relates to exploratory behaviors, and that dopaminergic deficiency may underlie some of these behavioral anomalies. In the present study and in our recently published work [7], we have conducted electrophysiological experiments in an effort to better understand the synaptic mechanisms underlying the deficits in novelty acquisition and learning and memory impairments in *Nf1* OPG mice. Previously-published research by other investigators have shown that LTD is a form of hippocampal synaptic plasticity that may be importantly involved in novelty acquisition. For example, low frequency stimulation (LFS) during exploration of a novel environment in freely-moving rats has been reported to result in either LTD or enhancement of LTD in a strain-dependent manner, while exploration of a familiar environment did not produce new expression of LTD [21]. Moreover, environmental exploration of unfamiliar objects and/or familiar objects in new locations also facilitated LTD [22]. Similarly, mice with forebrain deletion of serum response factor exhibited LTD deficits in hippocampal slices, which were associated with impairments in immediate memory of novel contexts [31]. In addition, we have found LTD deficits in hippocampal slices from mice that were deficient for the early growth response gene 3, which showed abnormal responses to novelty and stress [12]. In light of these findings, we explored the possibility that *Nf1* OPG mice had impaired LTD using a hippocampal slice preparation. However, no abnormalities in LTD were demonstrated in *Nf1* OPG mice since intact LFS-induced LTD was demonstrated in slices from both *Nf1* OPG and control mice.

In contrast to the normal LTD observed in hippocampal slices from *Nf1* OPG mice, we have demonstrated recently [7] that these mice show reduced LTP from high frequency stimulation using the same hippocampal slice preparation, which is rescued by treatment with a D1 receptor agonist (SKF38393). In that same study we reported that retention deficits were observed in *Nf1* OPG mice during probe trials in the Morris water maze which were rescued by administration of the same dose of L-dopa used in the current study. These LTP deficits in *Nf1* OPG mice suggest that reduced dompamine-mediated hippocampal neuronal function may play a role in the spatial learning/memory deficits found in these mice. Our behavioral and electrophysiological findings are consistent with those of Silva and colleagues who reported spatial learning/memory impairments and hippocampal LTP deficits in *Nf1* ^+/−^ mice, although they focused on different neurofibromin signaling pathways and neurotransmitters to explain their findings [32], [33]. The presence of hippocampal LTP deficits in *Nf1* OPG mice may also have relevance to the present results in that LTP has been implicated in some aspects of novelty acquisition [34]. While late phase- (late-) LTD in the Schaffer collateral-CA1 pathway of freely-moving rats is enhanced by object investigation, exploration of an empty novel environment facilitates late LTP [21], [22]. Relevant to studies using *Nf1* OPG mice, D1/D5 dopamine receptor antagonists have been reported to block: object-configuration learning; the enhancement of late-LTD by object investigation; and the induction of late-LTP following exploration of empty space [34]. In addition, antagonism of D1/D5 receptors can preclude late-LTP induced by electrical stimulation, while activation of dopamine receptors facilitates LTP and LTD induction by patterned electrical stimulation [34]. Thus, results from our present and previous work [5], [6], [7] raise the possibility that defective dopamine-based LTP processes may also underlie disturbances in novelty acquisition and related attention system processes, which may play some role in the spatial learning/memory deficits in *Nf1* OPG mice.

The results from our present and previous studies demonstrate that *Nf1* OPG mice have a complex behavioral phenotype. In the present report, we have focused on abnormal responses to novel environmental stimuli and other aberrant motivational influences in *Nf1* OPG mice. Characterizing these behavioral anomalies as well as understanding the underlying mechanisms have significance for several behavioral domains affected in children with NF1. It has been hypothesized that the formation of associative spatial memories through hippocampal LTP-like mechanisms requires exploration of the environment to first learn novel versus familiar contexts, which may require LTD-like processes [31]. In this regard, defining the interplay between LTP and LTD as it relates to novelty may have importance for dissecting the relative contributions of neurofibromin signaling pathway regulation {RAS, cyclic adenosine monophosphate (cAMP)} and neurochemical homeostasis {dopamine, gamma aminobutyric acid (GABA)} to the learning and memory deficits found in *Nf1* genetically-engineered mouse strains [5], [32], [33], [35]. Since LTD and LTP encode different aspects of novelty acquisition [22], understanding the relative contributions of each of these factors to cognitive performance in mice may yield more targeted approaches to drug therapies for children with NF1-associated learning and memory problems. Future research priorities focused on examining the complex interactions between Ras hyperactivation, lower cAMP levels, GABA inhibition, and dopamine neurotransmission in the hippocampus may reveal new opportunities for preclinical drug studies directed at improving cognitive disturbances in children with NF1.

## Supporting Information

Figure S1 **Performance of** ***Nf1*** **OPG and WT control mice on the elevated plus maze (EPM) and hanging object tests.** (A–D) In cohort 2, no significant main or interaction effects involving Genotype were found for entries made (A), time spent (B), percent of total arm distance traveled (C), or percent of total arm time spent (D) in the open arms of the EPM. (E) An rmANOVA revealed a significant Genotype by Test Day interaction for the percent of open arm entries made out of the total number of entries for both sets of arms, (††p = 0.024). Subsequent pair-wise comparisons showed that this effect was mostly due to differences observed during Test Day 3 (*p = 0.022) when the WT control mice made a greater percentage of entries into the open arms out of the total arm entries. (F) Planned comparisons showed that the WT control mice reared significantly more often to investigate the hanging object (ball) compared to levels of rearing exhibited in the opposite area of the field (*p = 0.001) while the rearing frequency in the two areas was not significantly different in the *Nf1* OPG group for the first hanging object test (cohort 2). The cohort 2 groups were 4.5–5.5 months old at testing and the sample sizes and sex distribution were the same for each group (n = 10: M = 4; F = 6). (G) No significant differences in ambulatory activity were observed among the CON+SAL, *Nf1* OPG+SAL, *Nf1* OPG+LDOPA groups during the second hanging object test (cohort 3). The male mice in cohort 3 were 3.5–4.5 months of age and each of the groups had the same sample size (n = 12).
(EPS)

Table S1 **ANOVA effects: Y-maze spontaneous alternations; elevated plus maze (EPM) variables (time, entries, %total arm entries, and %total arm time in open arms).**
(DOC)

Table S2 **ANOVA effects for elevated plus maze distance variables.**
(DOC)

Table S3 **ANOVA effects for first holeboard exploration/olfactory preference test (cohort 2).**
(DOC)

Table S4 **ANOVA effects for the first open-field test (cohort 2).**
(DOC)

Table S5 **ANOVA effects for first hanging object test (cohort 2).**
(DOC)

Table S6 **ANOVA effects for second holeboard exploration/olfactory preference test which included L-dopa administration (cohort 3).**
(DOC)

Table S7 **ANOVA effects for second open-field (OF) and hanging object (HO) tests which included L-dopa administration (cohort 3).**
(DOC)

## References

[1] Gutmann DH, Aylsworth A, Carey JC Korf B, Marks J, et al (1997) The diagnostic evaluation and multidisciplinary management of neurofibromatosis 1 and neurofibromatosis 2. JAMA 278: 51–57. 9207339

[2] Hyman SL, Shores A, North KN (2005) The nature and frequency of cognitive deficits in children with neurofibromatosis 1: a behavioral phenotype. Neurology 65: 1037–1044. 16217056 10.1212/01.wnl.0000179303.72345.ce

[3] Hyman SL, Shores A, North KN (2006) Learning disabilities in children with neurofibromatosis type 1: subtypes, cognitive profile, and attention-deficit-hyperactivity disorder. Dev Med Child Neurol 48: 973–977. 17109785 10.1017/S0012162206002131

[4] North KN, Riccardi V, Samango-Sprouse C, Ferner R, Moore B, et al (1997) Cognitive function and academic performance in neurofibromatosis 1: consensus statement from the NF1 Cognitive Disorders Task Force. Neurology 48: 1121–1127. 9109916 10.1212/wnl.48.4.1121

[5] Brown JA, Emnett RJ, White CR, Yuede C, Conyers SB, et al (2010) Reduced striatal dopamine underlies the attention system dysfunction in neurofibromatosis-1 mutant mice. Hum Mol Genet 19: 4515–4528. 20826448 10.1093/hmg/ddq382 PMC2957316

[6] Brown JA, Xu J, Diggs-Andrews KA, Wozniak DF, Mach RH, et al (2011) PET imaging for attention deficit preclinical drug testing in neurofibromatosis-1 mice. Exp Neurol 232: 333–338. 21963652 10.1016/j.expneurol.2011.09.005 PMC3202049

[7] Diggs-Andrews KA, Tokuda K, Izumi Y, Zorumski CF, Wozniak DF, et al (2012) Dopamine deficiency underlies learning deficits in neurofibromatosis-1 mice. Ann Neurol 73: 309–315. 23225063 10.1002/ana.23793 PMC3608728

[8] Mautner VF, Kluwe L, Thakker SD, Leark RA (2002) Treatment of ADHD in neurofibromatosis type 1. Dev Med Child Neurol 44: 164–170. 12005317 10.1017/s0012162201001876

[9] Brannan CL, Perkins AS, Vogel KS, Ratner N, Nordlund ML, et al (1994) Targeted disruption of the neurfibromatosis type-1 gene leads to developmental abnormalities in heart and various crest-derived tissues. Genes Dev 8: 1019–29. 7926784 10.1101/gad.8.9.1019

[10] Zhu Y, Romero MI, Ghosh P, Ye Z, Charnay P, et al (2001) Ablation of NF1 function in neurons induces abnormal development of cerebral cortex and reactive gliosis in brain. Genes Dev 15: 859–876. 11297510 10.1101/gad.862101 PMC312666

[11] Bajeneru ML, Hernandez MR, Perry A, Zhu Y, Parada LF, et al (2003) Optic nerve glioma in mice requires astrocyte gene inactivation and Nf1 brain heterozygosity. Cancer Res 63: 8573–8577. 14695164

[12] Gallitano-Mendel A, Izumi Y, Tokuda K, Zorumski CF, Muglia LJ, et al (2007) The immediate early gene early growth response gene 3 mediates adaptation to stress and novelty. Neuroscience 148: 633–643. 17692471 10.1016/j.neuroscience.2007.05.050 PMC2597331

[13] Schaefer ML, Wong ST, Wozniak DF, Muglia LM, Liauw JA, et al. Altered stress-induced anxiety in adenylyl cyclase type VIII-deficient mice. J Neurosci 20: 4809–4820. 10864938 10.1523/JNEUROSCI.20-13-04809.2000 PMC6772287

[14] Ghoshal N, Dearborn JT, Wozniak DF, Cairns NJ (2012) Core features of frontotemporal dementia recapitulated in progranulin knockout mice. Neurobiol Dis 45: 395–408. 21933710 10.1016/j.nbd.2011.08.029 PMC3225509

[15] Sato C, Turkoz M, Dearborn JT, Wozniak DF, Kopan R, et al (2012) Loss of RBPj in postnatal excitatory neurons does not cause neurodegeneration or memory impairments in aged mice. PLoS ONE 7(10): e48180 doi:10.1371/journal.pone.0048180 23110206 10.1371/journal.pone.0048180 PMC3482205

[16] Dougherty JD, Maloney SE, Wozniak DF, Rieger MA, Sonnenblick L, et al (2013) The disruption of Celf6, a gene identified by translational profiling of serotonergic neurons, results in autism-related behaviors. J Neurosci 33: 2732–2753. 23407934 10.1523/JNEUROSCI.4762-12.2013 PMC3711589

[17] Brosnan-Watters G, Wozniak DF (1997) A rotating holeboard procedure for testing drug effects on spatial learning and memory in mice. Brain Res Protoc 1: 331–338. 10.1016/s1385-299x(97)00007-x 9384812

[18] Wozniak DF, Hartman RE, Boyle MP, Vogt SK, Brooks AR, et al (2004) Apoptotic neurodegeneration induced by ethanol in neonatal mice is associated with profound learning/memory deficits in juveniles followed by progressive functional recovery in adults. Neurobiol Dis 17: 403–414. 15571976 10.1016/j.nbd.2004.08.006

[19] Wozniak DF, Xiao M, Xu L, Yamada KA, Ornitz DM (2007) Impaired spatial learning and defective theta burst induced LTP in mice lacking fibroblast growth factor 14. Neurobiol Dis 26: 14–26. 17236779 10.1016/j.nbd.2006.11.014 PMC2267915

[20] Lalonde R (2002) The neurobiological basis of spontaneous alternation. Neurosci Biobehav Rev 26: 91–104. 11835987 10.1016/s0149-7634(01)00041-0

[21] Mananhan-Vaughan D, Braunewell K–H (1999) Novelty acquisition is associated with induction of hippocampal long-term depression. Proc Natl Acad Sci USA 96: 8739–8744. 10411945 10.1073/pnas.96.15.8739 PMC17586

[22] Kemp A, Mananhan-Vaughan D (2004) Hippocampal long-term depression and long-term potentiation encode different aspects of novelty acquisition. Proc Natl Acad Sci USA 101: 8192–8197. 15150407 10.1073/pnas.0402650101 PMC419579

[23] Nakao K, Ikegaya Y, Yamada MK, Nishiyama N, Matsuki N (2002) Hippocampal long-term depression as an index of spatial working memory. Eur J Neurosci 16: 970–974. 12372034 10.1046/j.1460-9568.2002.02159.x

[24] File SE, Wardill AG (1975) Validity of head-dipping as a measure of exploration in a modified hole-board. Psychopharmacologia 44: 53–59. 1197580 10.1007/BF00421184

[25] Cave JW, Baker H (2009) Dopamine systems in the forebrain. Adv Exp Med Biol 651: 15–35. 19731547 10.1007/978-1-4419-0322-8_2 PMC2779115

[26] Doty RL (2012) Olfaction in Parkinson’s disease and related disorders. Neurobiol Dis 46: 527–552. 22192366 10.1016/j.nbd.2011.10.026 PMC3429117

[27] Schecklmann M, Schwenck C, Taurines R, Freitag C, Warnke A, et al (2013) A systematic review on olfaction in child and adolescent psychiatric disorders. J Neural Transm 120: 121–130. 22806003 10.1007/s00702-012-0855-2

[28] Moy SS, Nadler JJ, Poe MD, Nonneman RJ, Young NB, et al (2008) Development of a mouse test for repetitive, restricted behaviors: relevance to autism. Behav Brain Res 188: 178–194. 18068825 10.1016/j.bbr.2007.10.029 PMC2349090

[29] Aspied R, Fresiello A, de Filippis G, GironieCarnevale UA Sadile AG (2000) Nonselective attention in a rat model of hyperactivity and attention deficit: subchronic methylphenidate and nitric oxide synthesis inhibitor treatment. Neurosci Biobehav Rev 24: 59–71. 10654662 10.1016/s0149-7634(99)00045-7

[30] Vallone D, Pignatelli M, Grammatikopoulos G, Ruocco L, Bozzi Y, et al (2002) Activity, non-selective attention and emotionality in dopamine D2/D3 receptor knock-out mice. Behav Brain Res 130: 141–148. 11864730 10.1016/s0166-4328(01)00428-4

[31] Etkin A, Llarcon JM, Weisberg SP, Touzani K, Huang YY, et al (2006) A role in learning for SRF: deletion in the adult forebrain disrupts LTD and the formation of immediate memory of a novel context. Neuron 50: 127–143. 16600861 10.1016/j.neuron.2006.03.013

[32] Cui Y, Costa RM, Murphy GG, Elgersma Y, Zhu Y, et al (2008) Neurofibromin regulation of ERK signaling modulates GABA release and learning. Cell 135: 549–560. 18984165 10.1016/j.cell.2008.09.060 PMC2673196

[33] Li W, Cui Y, Kushner SA, Brown RA, Jentsch JD, et al (2005) The HMG-CoA reductase inhibitor lovastatin reverses the learning and attention deficits in a mouse model of neurofibromatosis type 1. Curr Biol 15: 1961–1967. 16271875 10.1016/j.cub.2005.09.043

[34] Lemon N, Manahan-Vaughan D (2006) Dopamine D1/D2 receptors gate the acquisition of novel information through hippocampal long-term potentiation and long-term depression. J Neurosci 26: 7723–7729. 16855100 10.1523/JNEUROSCI.1454-06.2006 PMC6674280

[35] Guilding C, McNair K, Stone TW, Morris BJ (2007) Restored plasticity in a mouse model of neurofibromatosis type 1 via inhibition of hyperactive ERK and CREB. Eur J Neurosci 25: 99–105. 17241271 10.1111/j.1460-9568.2006.05238.x

