# Supplementary material for: Motivational Disturbances and Effects of L-dopa Administration in Neurofibromatosis-1 Model Mice
Source: PLoS One. 2013 Jun 10;8(6):e66024. doi: 10.1371/journal.pone.0066024 (PMC3677926; doi:10.1371/journal.pone.0066024)
Supplement: Table S1 — ANOVA effects: Y-maze spontaneous alternations; elevated plus maze (EPM) variables (time, entries, %total arm entries, and %total arm time in open arms). (DOC) [file pone.0066024.s002.doc]

| **Table S1. ANOVA effects: Y-maze spontaneous alternations; elevated plus maze** | | |
| --- | --- | --- |
| (EPM) variables (time, entries, %total arm entries, and %total arm time in open arms). | | |
|  |  |  |
| Test/Variable | Effect |  |
|  |  |  |
| Y-Maze: Alternations |  |  |
|  | Genotype (Geno) | F(1,33)=8.11, p=0.008 |
|  | Sex | F(1,33)=0.03, p=0.86 |
|  | Geno x Sex | F(1,33)=0.25, p=0.62 |
|  |  |  |
| Y-Maze: Arm Entries |  |  |
|  | Genotype (Geno) | F(1,33)=7.24, p=0.011 |
|  | Sex | F(1,33)=0.08, p=0.77 |
|  | Geno x Sex | F(1,33)=0.003, p=0.96 |
|  |  |  |
| Y-Maze: %Alternations |  |  |
|  | Genotype (Geno) | F(1,33)=0.08, p=0.77 |
|  | Sex | F(1,33)=3.07, p=0.09 |
|  | Geno x Sex | F(1,33)=2.91, p=0.10 |
|  |  |  |
| EPM: Open Arm Time |  |  |
|  | Genotype (Geno) | F(1,16)=0.33, p=0.58 |
|  | Sex | F(1,16)=0.07, p=0.80 |
|  | Geno x Sex | F(1,16)=0.01, p=0.92 |
|  | Test Day (TD) | F(2,32)=8.02, p=0.006 |
|  | Geno xTD | F(2,32)=1.17, p=0.31 |
|  | Sex x TD | F(2,32)=0.95, p=0.37 |
|  | Geno x Sex x TD | F(2,32)=0.27, p=0.68 |
|  |  |  |
| EPM: Open Arm Entries |  |  |
|  | Genotype (Geno) | F(1,16)=0.24, p=0.63 |
|  | Sex | F(1,16)=0.16, p=0.70 |
|  | Geno x Sex | F(1,16)=1.82, p=0.20 |
|  | Test Day (TD) | F(2,32)=11.78, p=0.0007 |
|  | Geno xTD | F(2,32)=0.98, p=0.37 |
|  | Sex x TD | F(2,32)=2.07, p=0.16 |
|  | Geno x Sex x TD | F(2,32)=1.30, p=0.28 |
|  |  |  |
| EPM: %Open Arm Time |  |  |
|  | Genotype (Geno) | F(1,16)=0.40, p=0.54 |
|  | Sex | F(1,16)=0.04, p=0.84 |
|  | Geno x Sex | F(1,16)=0.001, p=0.97 |
|  | Test Day (TD) | F(2,32)=8.12, p=0.005 |
|  | Geno xTD | F(2,32)=1.11, p=0.33 |
|  | Sex x TD | F(2,32)=0.87, p=0.40 |
|  | Geno x Sex x TD | F(2,32)=0.22, p=0.73 |
|  |  |  |
| EPM: %Open Arm Entries |  |  |
|  | Genotype (Geno) | F(1,16)=0.0007, p=0.98 |
|  | Sex | F(1,16)=0.40, p=0.54 |
|  | Geno x Sex | F(1,16)=1.09, p=0.31 |
|  | Test Day (TD) | F(2,32)=6.78, p=0.004 |
|  | Geno xTD | F(2,32)=4.31, p=0.024 |
|  | Sex x TD | F(2,32)=3.24, p=0.055 |
|  | Geno x Sex x TD | F(2,32)=0.07, p=0.93 |
