# Supplementary material for: Motivational Disturbances and Effects of L-dopa Administration in Neurofibromatosis-1 Model Mice
Source: PLoS One. 2013 Jun 10;8(6):e66024. doi: 10.1371/journal.pone.0066024 (PMC3677926; doi:10.1371/journal.pone.0066024)
Supplement: Table S2 — ANOVA effects for elevated plus maze distance variables. (DOC) [file pone.0066024.s003.doc]

| **Table S2. ANOVA effects for elevated plus maze distance variables.** | | |
| --- | --- | --- |
|  |  |  |
| Test/Variable | Effect |  |
|  |  |  |
| Open Arm Distance |  |  |
|  | Genotype (Geno) | F(1,16)=0.19, p=0.67 |
|  | Sex | F(1,16)=0.20, p=0.66 |
|  | Geno x Sex | F(1,16)=0.22, p=0.65 |
|  | Test Day (TD) | F(2,32)=5.15, p=0.024 |
|  | Geno x TD | F(2,32)=1.55, p=0.23 |
|  | Sex x TD | F(2,32)=1.61, p=0.22 |
|  | Geno x Sex x TD | F(2,32)=0.21, p=0.73 |
|  |  |  |
| Center Distance |  |  |
|  | Genotype (Geno) | F(1,16)=1.27, p=0.93 |
|  | Sex | F(1,16)=0.01, p=0.66 |
|  | Geno x Sex | F(1,16)=1.08, p=0.31 |
|  | Test Day (TD) | F(2,32)=53.24, p<0.00005 |
|  | Geno x TD | F(2,32)=0.51, p=0.61 |
|  | Sex x TD | F(2,32)=2.81, p=0.08 |
|  | Geno x Sex x TD | F(2,32)=0.29, p=0.76 |
|  |  |  |
| Total Distance |  |  |
|  | Genotype (Geno) | F(1,16)=8.74, p=0.009 |
|  | Sex | F(1,16)=0.03, p=0.87 |
|  | Geno x Sex | F(1,16)=2.03, p=0.17 |
|  | Test Day (TD) | F(2,32)=33.71, p<0.00005 |
|  | Geno x TD | F(2,32)=0.96, p=0.38 |
|  | Sex x TD | F(2,32)=0.49, p=0.58 |
|  | Geno x Sex x TD | F(2,32)=0.62, p=0.51 |
|  | Test Day 1 | F(1,16)=3.31, p=0.09 |
|  | Test Day 2 | F(1,16)=7.47, p=0.015 |
|  | Test Day 3 | F(1,16)=8.88, p=0.009 |
|  |  |  |
| Closed Arm Distance |  |  |
|  | Genotype (Geno) | F(1,16)=14.60, p=0.0015 |
|  | Sex | F(1,16)=0.006, p=0.94 |
|  | Geno x Sex | F(1,16)=2.03, p=0.17 |
|  | Test Day (TD) | F(2,32)=11.54, p=0.0004 |
|  | Geno x TD | F(2,32)=0.22, p=0.77 |
|  | Sex x TD | F(2,32)=3.21, p=0.06 |
|  | Geno x Sex x TD | F(2,32)=0.76, p=0.46 |
|  | Test Day 1 | F(1,16)=6.19, p=0.024 |
|  | Test Day 2 | F(1,16)=12.46, p=0.003 |
|  | Test Day 3 | F(1,16)=9.83, p=0.006 |
|  |  |  |
| %Open Arm Distance |  |  |
|  | Genotype (Geno) | F(1,16)=0.50, p=0.67 |
|  | Sex | F(1,16)=0.41, p=0.53 |
|  | Geno x Sex | F(1,16)=0.07, p=0.80 |
|  | Test Day (TD) | F(2,32)=4.19, p=0.04 |
|  | Geno x TD | F(2,32)=2.16, p=0.15 |
|  | Sex x TD | F(2,32)=2.40, p=0.13 |
|  | Geno x Sex x TD | F(2,32)=0.03, p=0.92 |
