# Supplementary material for: Motivational Disturbances and Effects of L-dopa Administration in Neurofibromatosis-1 Model Mice
Source: PLoS One. 2013 Jun 10;8(6):e66024. doi: 10.1371/journal.pone.0066024 (PMC3677926; doi:10.1371/journal.pone.0066024)
Supplement: Table S3 — ANOVA effects for first holeboard exploration/olfactory preference test (cohort 2). (DOC) [file pone.0066024.s004.doc]

| **Table S3. ANOVA effects for first holeboard exploration/olfactory preference test.** | | |
| --- | --- | --- |
| (cohort 2). Pks=Pokes; Dur=Duration | | |
|  | | |
| Test/Variable | Effect |  |
|  |  |  |
| Hole Poke Frequencies |  |  |
|  |  |  |
| Total Hole Pokes |  |  |
|  | Genotype (Geno) | F(1,16)=6.35, p=0.023 |
|  | Sex | F(1,16)=0.12, p=0.73 |
|  | Geno x Sex | F(1,16)=1.84, p=0.19 |
|  |  |  |
| Side Hole Pokes |  |  |
|  | Genotype (Geno) | F(1,16)=3.91, p=0.066 |
|  | Sex | F(1,16)=0.10, p=0.81 |
|  | Geno x Sex | F(1,16)=1.23, p=0.28 |
|  |  |  |
| Total Ambulations |  |  |
|  | Genotype (Geno) | F(1,16)=4.37, p=0.053 |
|  | Sex | F(1,16)=0.06, p=0.81 |
|  | Geno x Sex | F(1,16)=0.004, p=0.95 |
|  |  |  |
| Empty vs Odor Corner Pks |  |  |
|  | Genotype (Geno) | F(1,16)=7.41, p=0.015 |
|  | Sex | F(1,16)=0.12, p=0.74 |
|  | Geno x Sex | F(1,16)=2.06, p=0.17 |
|  | Hole Type (HT) | F(1,16)=3.21, p=0.09 |
|  | Geno x HT | F(1,16)=1.62, p=0.22 |
|  | Sex x HT | F(1,16)=0.40, p=0.53 |
|  | Geno x Sex x HT | F(1,16)=0.33, p=0.57 |
|  |  |  |
| Novel vs Familiar Pks |  |  |
|  | Genotype (Geno) | F(1,16)=6.89, p=0.018 |
|  | Sex | F(1,16)=0.008, p=0.93 |
|  | Geno x Sex | F(1,16)=1.84, p=0.19 |
|  | Hole Type (HT) | F(1,16)=0.55, p=0.47 |
|  | Geno x HT | F(1,16)=0.37, p=0.55 |
|  | Sex x HT | F(1,16)=0.45, p=0.51 |
|  | Geno x Sex x HT | F(1,16)=0.65, p=0.43 |
|  |  |  |
| Hole Poke Durations |  |  |
|  |  |  |
| Empty vs Odor Poke Dur |  |  |
|  | Genotype (Geno) | F(1,9)=2.77, p=0.13 |
|  | Sex | F(1,9)=0.22, p=0.65 |
|  | Geno x Sex | F(1,9)=0.004, p=0.95 |
|  | Hole Type (HT) | F(1,9)=9.44, p=0.013 |
|  | Geno x HT | F(1,9)=0.40, p=0.54 |
|  | Sex x HT | F(1,9)=2.46, p=0.15 |
|  | Geno x Sex x HT | F(1,9)=1.50, p=0.25 |
|  |  |  |
|  |  |  |
|  |  |  |
|  |  |  |
|  |  |  |
