# Supplementary material for: Motivational Disturbances and Effects of L-dopa Administration in Neurofibromatosis-1 Model Mice
Source: PLoS One. 2013 Jun 10;8(6):e66024. doi: 10.1371/journal.pone.0066024 (PMC3677926; doi:10.1371/journal.pone.0066024)
Supplement: Table S4 — ANOVA effects for the first open-field test (cohort 2). (DOC) [file pone.0066024.s005.doc]

| **Table S4. ANOVA effects for the first open-field test (cohort 2).** | | |
| --- | --- | --- |
|  | | |
| Test/Variable | Effect |  |
|  |  |  |
| Open-Field: Total Ambulations |  |  |
|  | Genotype (Geno) | F(1,16)=5.93, p=0.027 |
|  | Sex | F(1,16)=1.04, p=0.32 |
|  | Geno x Sex | F(1,16)=0.58, p=0.46 |
|  | Time | F(2,32)=99.07, p<0.00005 |
|  | Geno x Time | F(2,32)=3.82, p=0.033 |
|  | Sex x Time | F(2,32)=2.54, p=0.09 |
|  | Geno x Sex x Time | F(2,32)=0.37, p=0.69 |
|  | Time Block 1 | F(1,16)=7.46, p=0.015 |
|  | Time Block 2 | F(1,16)=5.10, p=0.038 |
|  | Time Block 3 | F(1,16)=1.68, p=0.21 |
|  |  |  |
| Open-Field: Rearing Frequency |  |  |
|  | Genotype (Geno) | F(1,16)=2.06, p=0.17 |
|  | Sex | F(1,16)=0.12, p=0.73 |
|  | Geno x Sex | F(1,16)=1.21, p=0.29 |
|  | Time | F(2,32)=0.53, p=0.59 |
|  | Geno x Time | F(2,32)=3.47, p=0.043 |
|  | Sex x Time | F(2,32)=0.42, p=0.66 |
|  | Geno x Sex x Time | F(2,32)=0.04, p=0.96 |
|  | Time Block 1 | F(1,16)=8.62, p=0.010 |
|  | Time Block 2 | F(1,16)=1.93, p=0.18 |
|  | Time Block 3 | F(1,16)=0.0001, p=0.99 |
|  |  |  |
| Open-Field Rearing Time |  |  |
|  | Genotype (Geno) | F(1,16)=5.00, p=0.040 |
|  | Sex | F(1,16)=0.46, p=0.51 |
|  | Geno x Sex | F(1,16)=3.90, p=0.066 |
|  | Time | F(1,16)=0.74, p=0.48 |
|  | Geno x Time | F(1,16)=1.86, p=0.17 |
|  | Sex x Time | F(1,16)=0.30, p=0.74 |
|  | Geno x Sex x Time | F(1,16)=0.01, p=0.99 |
|  | Time Block 1 | F(1,16)=10.77, p=0.005 |
|  | Time Block 2 | F(1,16)=4.70, p=0.046 |
|  | Time Block 3 | F(1,16)=0.82, p=0.38 |
|  |  |  |
|  |  |  |
|  |  |  |
|  |  |  |
|  |  |  |
|  |  |  |
|  |  |  |
|  |  |  |
|  |  |  |
|  |  |  |
|  |  |  |
|  |  |  |
|  |  |  |
|  |  |  |
