# Supplementary material for: Motivational Disturbances and Effects of L-dopa Administration in Neurofibromatosis-1 Model Mice
Source: PLoS One. 2013 Jun 10;8(6):e66024. doi: 10.1371/journal.pone.0066024 (PMC3677926; doi:10.1371/journal.pone.0066024)
Supplement: Table S5 — ANOVA effects for first hanging object test (cohort 2). (DOC) [file pone.0066024.s006.doc]

| **Table S5. ANOVA effects for first hanging object test (cohort 2).** | | |
| --- | --- | --- |
|  | | |
| Test/Variable | Effect |  |
|  |  |  |
| Rearing Time For Object |  |  |
|  | Genotype (Geno) | F(1,16)=6.34, p=0.023 |
|  | Sex | F(1,16)=0.07, p=0.79 |
|  | Geno x Sex | F(1,16)=0.73, p=0.41 |
|  | Area (Ball vs Opposite) | F(1,16)=17.42, p=0.0007 |
|  | Geno x Area | F(1,16)=8.59, p=0.010 |
|  | Sex x Area | F(1,16)=0.03, p=0.87 |
|  | Geno x Sex x Area | F(1,16)=0.28, p=0.61 |
|  | Ball | F(1,16)=7.68, p=0.014 |
|  | Opposite Area (OPP) | F(1,16)=2.09, p=0.17 |
|  | *Nf1* OPG: OPP vs Ball | F(1,16)=0.77, p=0.39 |
|  | Con: OPP vs Ball | F(1,16)=25.23, p=0.0001 |
|  |  |  |
| Rearing Frequency For Object |  |  |
|  | Genotype (Geno) | F(1,16)=2.24, p=0.091 |
|  | Sex | F(1,16)=0.63, p=0.44 |
|  | Geno x Sex | F(1,16)=0.10, p=0.75 |
|  | Area | F(1,16)=15.37, p=0.0012 |
|  | Geno x Area | F(1,16)=2.48, p=0.014 |
|  | Sex x Area | F(1,16)=0.52, p=0.48 |
|  | Geno x Sex x Area | F(1,16)=0.26, p=0.62 |
|  | Ball | F(1,16)=3.23, p=0.09 |
|  | Opposite Area (OPP) | F(1,16)=1.70, p=0.21 |
|  | *Nf1* OPG: OPP vs Ball | F(1,16)=2,75, p=0.12 |
|  | Con: OPP vs Ball | F(1,16)=15.10, p=0.001 |
|  |  |  |
| Total Rearing Time In Field |  |  |
|  | Genotype (Geno) | F(1,16)=7.70, p=0.014 |
|  | Sex | F(1,16)=0.26, p=0.62 |
|  | Geno x Sex | F(1,16)=2.15, p=0.16 |
|  |  |  |
| % Total Rearing Time - Object |  |  |
|  | Genotype (Geno) | F(1,13)=3,46, p=0.086 |
|  | Sex | F(1,13)=0.06, p=0.81 |
|  | Geno x Sex | F(1,13)=0.008, p=0.93 |
|  | Area | F(1,13)=11.75, p=0.005 |
|  | Geno x Area | F(1,13)=3.57, p=0.08 |
|  | Sex x Area | F(1,13)=0.08, p=0.78 |
|  | Geno x Sex x Area | F(1,13)=0.16, p=0.69 |
|  | Ball | F(1,13)=3.70, p=0.08 |
|  | Opposite Area (OPP) | F(1,13)=0.001, p=0.97 |
|  | *Nf1* OPG: OPP vs Ball | F(1,13)=0.89, p=0.36 |
|  | Con: OPP vs Ball | F(1,13)=16.33, p=0.001 |
|  |  |  |
|  |  |  |
|  |  |  |
|  |  |  |
|  |  |  |
|  |  |  |
|  |  |  |
