# Supplementary material for: Motivational Disturbances and Effects of L-dopa Administration in Neurofibromatosis-1 Model Mice
Source: PLoS One. 2013 Jun 10;8(6):e66024. doi: 10.1371/journal.pone.0066024 (PMC3677926; doi:10.1371/journal.pone.0066024)
Supplement: Table S6 — ANOVA effects for second holeboard exploration/olfactory preference test which included L-dopa administration (cohort 3). (DOC) [file pone.0066024.s007.doc]

| **Table S6. ANOVA effects for second holeboard exploration/olfactory preference** | | |
| --- | --- | --- |
| test which included L-dopa administration (cohort 3). Pks=Pokes; Dur=Duration | | |
|  | | |
| Test/Variable | Effect |  |
|  |  |  |
| Hole Poke Frequencies |  |  |
|  |  |  |
| Total Hole Pokes |  |  |
|  | Group | F(2,33)=0.27, p=0.76 |
|  |  |  |
| Side Hole Pokes |  |  |
|  | Group | F(2,33)=0.35, p=0.71 |
|  |  |  |
| Total Ambulations |  |  |
|  | Group | F(2,33)=0.12, p=0.89 |
|  |  |  |
| Empty vs Odor Corner |  |  |
|  | Group | F(2,33)=0.28, p=0.76 |
|  | Hole Type | F(1,33)=15.67, p=0.0004 |
|  | Group x Hole Type | F(2,33)=1.16, p=0.33 |
|  | Odor (OD) | F(2,33)=0.52, p=0.60 |
|  | Empty (Em) | F(2,33)=0.53, p=0.59 |
|  | *Nf1* OPG+Sal: OD vs Em | F(1,33)=1.29, p=0.26 |
|  | *Nf1* OPG+L-Dopa: OD vs Em | F(1,33)=5.99, p=0.020 |
|  | Con+Sal: OD vs Em | F(1,33)=10.72, p=0.003 |
|  |  |  |
| Novel vs Familiar Odor |  |  |
|  | Group | F(2,33)=0.52, p=0.60 |
|  | Hole Type | F(1,33)=42,14, p<0.00005 |
|  | Group x Hole Type | F(2,33)=4.45, p=0.019 |
|  | Familiar (Fam) | F(2,33)=1.58, p=0.22 |
|  | Novel (Nov) | F(2,33)=3.29, p=0.0497 |
|  | *Nf1* OPG+Sal: Fam vs Nov | F(1,33)=1.79, p=0.19 |
|  | *Nf1* OPG+L-Dopa: Fam vs Nov | F(1,33)=27.72, p<0.00005 |
|  | Con+Sal: Fam vs Nov | F(1,33)=21.53, p=0.0001 |
|  |  |  |
| Hole Poke Durations |  |  |
|  |  |  |
| Empty vs Odor |  |  |
|  | Group | F(2,33)=2.17, p=0.13 |
|  | Hole Type | F(1,33)=19.19, p=0.0001 |
|  | Group x Hole Type | F(2,33)=0.85, p=0.44 |
|  | Odor (OD) | F(2,33)=1.88, p=0.17 |
|  | Empty (Em) | F(2,33)=1.40, p=0.59 |
|  | *Nf1* OPG+Sal: OD vs Em | F(1,33)=3.54, p=0.07 |
|  | *Nf1* OPG+L-Dopa: OD vs Em | F(1,33)=4.51, p=0.041 |
|  | Con+Sal: OD vs Em | F(1,33)=12.84, p=0.001 |
|  |  |  |
|  |  |  |
|  |  |  |
|  |  |  |
|  |  |  |
|  |  |  |
