# Supplementary material for: Motivational Disturbances and Effects of L-dopa Administration in Neurofibromatosis-1 Model Mice
Source: PLoS One. 2013 Jun 10;8(6):e66024. doi: 10.1371/journal.pone.0066024 (PMC3677926; doi:10.1371/journal.pone.0066024)
Supplement: Table S7 — ANOVA effects for second open-field (OF) and hanging object (HO) tests which included L-dopa administration (cohort 3). (DOC) [file pone.0066024.s008.doc]

| **Table S7. ANOVA effects for second open-field (OF) and hanging object (HO) tests** | | |
| --- | --- | --- |
| which included L-dopa administration (cohort 3). Opp= Opposite Area | | |
|  | | |
| Test/Variable | Effect |  |
|  |  |  |
| OF: Total Ambulations |  |  |
|  | Group | F(2,33)=0.63, p=0.54 |
|  | Time | F(2,66)=19.43, p<0.00005 |
|  | Group x Time | F(4,66)=1.83, p=0.13 |
|  | Time Block 1 | F(2,33)=0.06, p=0.94 |
|  | Time Block 2 | F(2,33)=1.93, p=0.16 |
|  | Time Block 3 | F(2,33)=0.34, p=0.71 |
|  |  |  |
| OF: Rearing |  |  |
|  | Group | F(2,33)=0.53, p=0.59 |
|  | Time | F(2,66)=4.95, p=0.01 |
|  | Group x Time | F(4,66)=1.89, p=0.12 |
|  | Time Block 1 | F(2,33)=0.55, p=0.58 |
|  | Time Block 2 | F(2,33)=1.32, p=0.28 |
|  | Time Block 3 | F(2,33)=0.19, p=0.82 |
|  |  |  |
| OF: Time Spent Rearing |  |  |
|  | Group | F(2,33)=0.77, p=0.47 |
|  | Time | F(2,66)=7.04, p=0.002 |
|  | Group x Time | F(4,66)=2.02, p=0.11 |
|  | Time Block 1 | F(2,33)=1.01, p=0.38 |
|  | Time Block 2 | F(2,33)=1.42, p=0.26 |
|  | Time Block 3 | F(2,33)=0.38, p=0.69 |
|  |  |  |
| HO: Rearing Time |  |  |
|  | Group | F(2,33)=1.23, p=0.30 |
|  | Area (Ball vs Opposite) | F(1,33)=7.70, p=0.009 |
|  | Group x Area | F(2,33)=1.23, p=0.31 |
|  | *Nf1* OPG+Sal: Ball vs Opp | F(1,33)=0.14, p=0.71 |
|  | *Nf1* OPG+L-Dopa: Ball vs Opp | F(1,33)=3.67, p=0.064 |
|  | Con+Sal: Ball vs Opp | F(1,33)=6.36, p=0.017 |
|  |  |  |
| HO: Rearing Frequency |  |  |
|  | Group | F(2,33)=1.19, p=0.32 |
|  | Area (Ball vs Opposite) | F(1,33)=8.96, p=0.005 |
|  | Group x Area | F(2,33)=1.23, p=0.30 |
|  | *Nf1* OPG+Sal: Ball vs Opp | F(1,33)=0.28, p=0.60 |
|  | *Nf1* OPG+L-Dopa: Ball vs Opp | F(1,33)=3.72, p=0.063 |
|  | Con+Sal: Ball vs Opp | F(1,33)=7.43, p=0.010 |
|  |  |  |
| HO: Total Rearing Time |  |  |
|  | Group | F(2,33)=0.87, p=0.43 |
|  |  |  |
| HO: Total Ambulations |  |  |
|  | Group | F(2,33)=0.52, p=0.60 |
|  |  |  |
|  |  |  |
|  |  |  |
|  |  |  |
|  |  |  |
